# Gold(I)-catalysed synthesis of a furan analogue of thiamine pyrophosphate

**DOI:** 10.3762/bjoc.10.270

**Published:** 2014-11-05

**Authors:** Amjid Iqbal, El-Habib Sahraoui, Finian J Leeper

**Affiliations:** 1 University of Cambridge, Department of Chemistry, Lensfield Road, Cambridge, CB2 1EW, U.K.

**Keywords:** furan synthesis, gold-catalysed cyclisation, pyruvate decarboxylase, thiamine diphosphate

## Abstract

An analogue of thiamine having a furan ring in place of the thiazolium ring has been synthesised by a short and efficient route, involving gold(I)-catalysed cyclisation of an alkynyl alcohol to form the furan ring. The furan analogue of thiamine diphosphate (ThDP) was also made and tested for binding to and inhibition of pyruvate decarboxylase (PDC) from *Zymomonas mobilis* (overexpressed in *E. coli* with a N-terminal His-tag). It is a very strong inhibitor, with a *K*_i_ value of 32.5 pM. It was also shown that the furan analogue of thiamine can be functionalised at the C-2 position, which will allow access to mimics of reaction intermediates of various ThDP-dependent enzymes.

## Introduction

**Figure 1:**
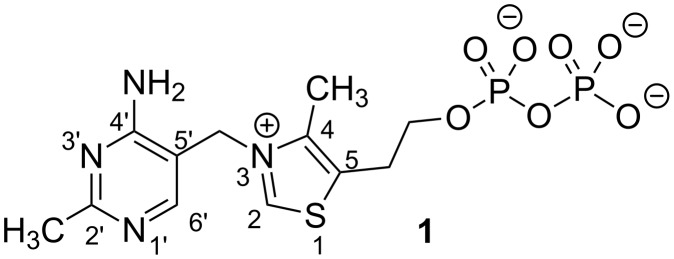
Structure of thiamine diphosphate (ThDP, **1**).

The biologically active form of vitamin B_1_ is thiamine diphosphate (ThDP, **1**, Figure 1), which is an essential cofactor and involved in a number of metabolic pathways, including oxidative and non-oxidative decarboxylation of α-keto acids (e.g., pyruvate dehydrogenase, pyruvate decarboxylase), the formation of amino acid precursors (acetohydroxy acid synthase), and ketol transfer between sugars (transketolase) [1]. One common feature of ThDP-dependent enzymes is to catalyse the cleavage and formation of bonds adjacent to the carbon of a carbonyl group with the thiazolium ring of ThDP acting as an electron sink during catalysis in order to stabilise what would otherwise be an acyl carbanion in the form of an enamine intermediate [2].

**Scheme 1:**
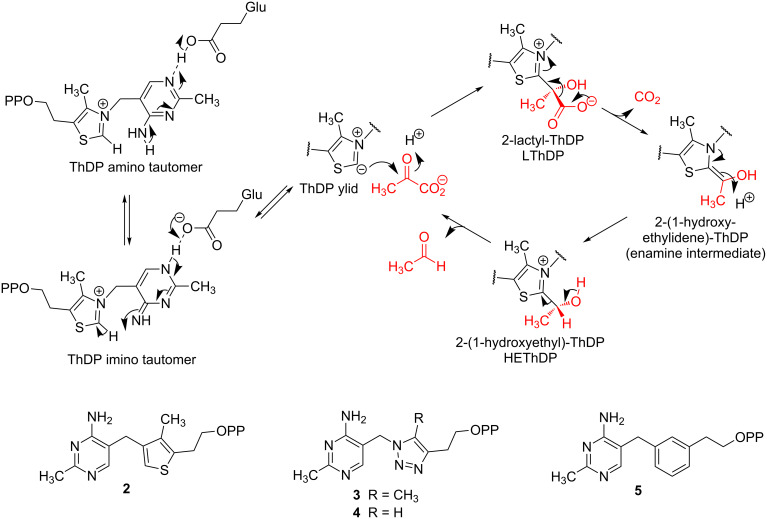
Mechanism of pyruvate decarboxylase and structures of some previously synthesised ThDP analogues.

The catalytic cycle for pyruvate decarboxylase (PDC, a representative ThDP-dependent enzyme), first proposed by Breslow [3–4], is given in Scheme 1. After formation of the ThDP ylid, the keto group of the substrate is attacked by the thiazolium C-2 carbanion to form the tetrahedral pre-decarboxylation intermediate 2-(2-lactyl)-ThDP, LThDP. It has been proposed that the covalent attachment of pyruvate to form LThDP introduces significant strain, the release of which is a driving force for the decarboxylation [5]. The effects of this type of strain on bond angles and lengths have recently been observed in a high resolution crystal structure of another ThDP-dependent enzyme, transketolase [6]. Another important factor of this decarboxylation is the presence of an electron sink in the form of the positively charged nitrogen atom. Decarboxylation gives the C-2α-carbanion/enamine resonance forms of the post decarboxylation intermediate. Protonation of this enamine intermediate at C-2α gives HEThDP and final elimination of acetaldehyde completes the catalytic cycle.

The 4’-aminopyrimidine ring of ThDP can interconvert among three ionization/tautomeric states, the protonated form and two neutral forms, the amino tautomer and the imino tautomer (Scheme 1) and it is believed to be the imine nitrogen atom of the latter that is the base that deprotonates C-2 to form the ylid [7–8].

Although many crystal structures of ThDP dependent enzymes exist, there still are some unanswered questions regarding mechanistic processes of these enzymes (e.g., how the substrate binds, what is the role of important catalytic groups, how are individual steps accelerated). In order to answer these questions, it would help to have snapshots of the enzyme bound to various reaction intermediates in the cycle. However, this is usually not possible when using the natural cofactor because the enzyme turns over rapidly, which in most cases prevents the trapping of reaction intermediates bound to the enzyme. One solution to this problem is to use analogues of ThDP that closely mimic the reaction intermediates of the catalytic cycle, but crucially are unable to continue the reaction mechanism. To this end we have previously synthesised inactive analogues of the ThDP cofactor, including 3-deazaThDP **2**, in which the nitrogen atom of the thiazolium ring is replaced by a carbon [9–10]. 3-DeazaThDP is isoelectronic with ThDP, but the lack of positive charge means that deprotonation at C-2 is not possible.

3-DeazaThDP **2** is an almost irreversible inhibitor, which binds 25,000 times more strongly (*K*_i_ = 14 pM) than ThDP to PDC from *Zymomonas mobilis* (*Zm*PDC) and 500 times more strongly (*K*_i_ = 5 nM) to the E1 component of α-ketoglutarate dehydrogenase [10]. This extremely tight binding can be rationalised by the structural similarity between **2** and the ylid of ThDP, with increased hydrophobic interactions between the enzyme and the electrically neutral analogue compared to the positively charged ThDP [11].

The synthesis of **2** was achieved in 12 steps in reasonable yield but was lengthy and time-consuming. For this reason other analogues have also been studied. Two triazole analogues **3** (*K*_i_ = 20 pM) and **4** (*K*_i_ = 30 pM against *Zm*PDC) were synthesised in just four synthetic steps [12]. However, there is no possibility of attaching a substituent at the 2-position of the triazole to mimic enzymic reaction intermediates. Another analogue of ThDP **5** with a benzene ring in place of the thiazolium ring was synthesised and also found to be a very strong inhibitor of *Zm*PDC, binding at almost the same rate as **2** [10]. With this benzene analogue also, it would be difficult to functionalise the 2-position selectively.

In this paper, we report a short and efficient synthesis of a furan-based analogue of thiamine. This analogue was converted to its diphosphate, which is another extremely potent inhibitor of *Zm*PDC, and could also be functionalised at the 2-position using a Friedel–Crafts acylation.

## Results and Discussion

### Synthesis of ThDP analogue **17**

**Scheme 2:**
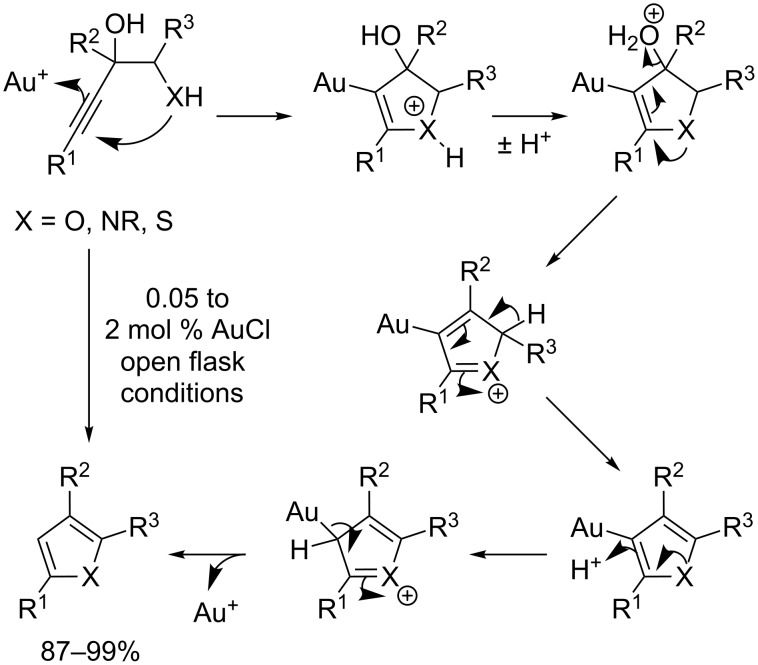
Dehydrative cyclization catalysed by gold(I) and its presumed mechanism [31].

The key step in our planned synthesis was the formation of the furan ring. Homogeneous gold-catalysed reactions have been used recently in the synthesis of furans from alkynes [13–20]. The ease with which alkynes, allenes and alkenes can be activated by Au(I) catalysts to form carbon–carbon and carbon–heteroatom bonds makes the method an appealing strategy towards diverse chemical targets [21–30]. Furthermore, the catalysts are stable to air and moisture and the reactions generally proceed under mild conditions. Aponick and co-workers reported a catalytic dehydrative cyclisation reaction of alkynyl alcohols catalysed by simple gold(I) salts [31]. The reaction proceeds rapidly under mild, open flask conditions to provide aromatic heterocycles such as furans, pyrroles and thiophenes in high yield with low catalyst loadings (Scheme 2).

**Scheme 3:**
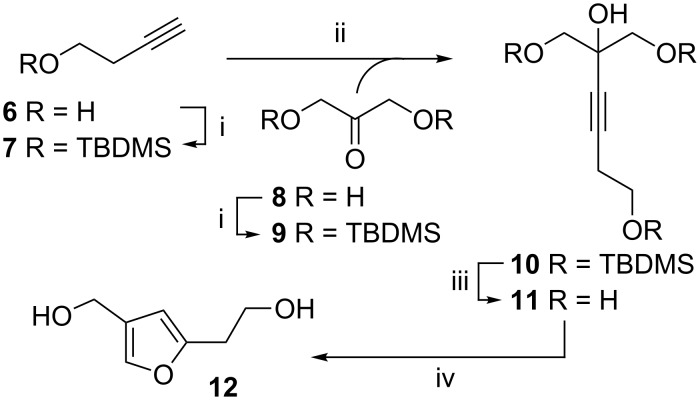
Synthesis of furan **12**. Reagents and conditions: (i) TBDMS-Cl, *N*-methylimidazole. (ii) *n*-BuLi, −78 °C, then **9** and to rt, 55% (iii) TBAF, THF, 79%. (iv) AuCl, THF, 85%.

Following this synthetic strategy, it was decided to explore the cyclisation of alkyne tetraol **11**, which should give furan **12** with hydroxyethyl and hydroxymethyl substituents at the 2- and 4-position respectively (Scheme 3). This could then be converted into a furan analogue of ThDP.

The first step in the synthesis of tetraol **11** was the protection of 3-butynol (**6**) and 1,3-dihydroxyacetone (**8**) as their TBDMS ethers, **7** and **9**. The alkyne **7** was then coupled with ketone **9** using *n*-BuLi. TBAF-mediated deprotection gave unsaturated alcohol **11**, the substrate for dehydrative cyclization with AuCl. The order of addition, reaction time and temperature were all found to be important for this reaction: the best yield was obtained when a solution of **11** in THF was added at 0 °C to the dry AuCl. The mixture was stirred for 10 min and then filtered through Celite to remove the catalyst. Using this method an 85% yield of furan **12** could be obtained. Delay in filtration or increase in temperature caused the catalyst to decompose and made the product difficult to purify. Subsequent to our synthesis of **12** a very similar synthesis of the same compound was published by Deslongchamps and coworkers [32], which only differed in that the cyclisation of **11** to **12** in excellent yield was catalysed by Hg(II), instead of Au(I).

**Scheme 4:**
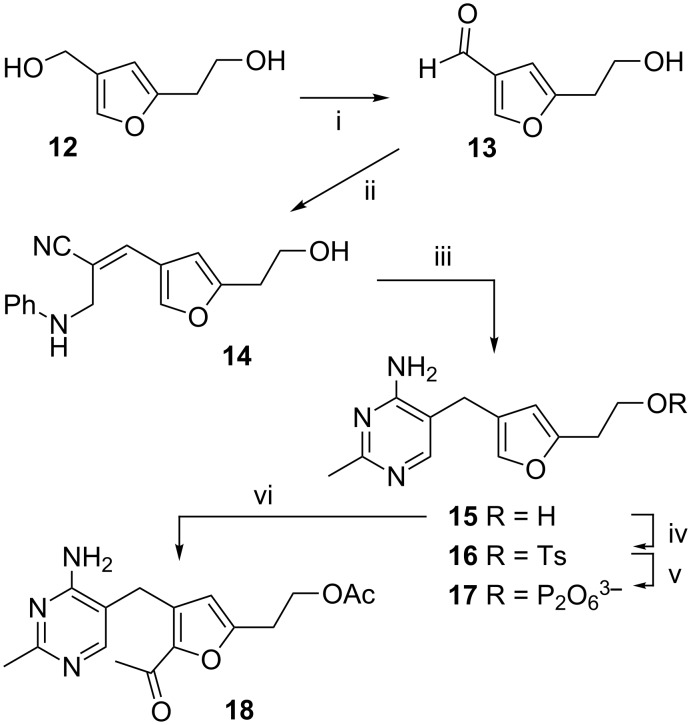
Synthesis of the furan analogue **17** of ThDP. Reagents and conditions: (i) MnO_2_, CHCl_3_, 72%; (ii) PhNHCH_2_CH_2_CN, NaOMe, DMSO, MeOH, 76%; (iii) CH_3_C(NH)NH_2_·HCl, NaOEt, EtOH, 65%; (iv) TsCl, pyridine, −5 °C, 72%; (v) TBA_3_HPP, MeCN, 4 °C, 30%; (vi) AcCl, AlCl_3_, DCM, 67%.

With furan **12** in hand, it was then transformed into the furan analogue **17** of ThDP by the same route as previously used for the synthesis of deazaThDP **2** [9–10], Scheme 4. Thus selective oxidation of the benzylic alcohol with MnO_2_, condensation with 3-anilinopropionitrile, and then reaction with acetamidine gives the thiamine analogue **15**. Tosylation of the alcohol and displacement of tosylate by the diphosphate trianion worked well to give ThDP analogue **17**.

A further objective was to show that the furan ring could be substituted at C-2. To this end furan **15** was reacted with acetyl chloride and AlCl_3_ and the 2-acetylfuran **18** was obtained in 67% yield. ^1^H,^13^C correlations in the HMBC spectrum of **18** proved that C-2 and not C-4 had been acetylated, with a key correlation being from the first CH_2_ of the CH_2_CH_2_OAc side-chain (C-5a) to the furan carbon that has a hydrogen attached (C-4). This selective acylation of C-2 will allow the addition of substituents at this position that match the substituents in the enzymic reactions, such as the 2-hydroxyethyl group found in HEThDP (Scheme 1).

### Inhibition of pyruvate decarboxylase by furan **17**

The furan analogue of ThDP, **17**, was tested as an inhibitor of pyruvate decarboxylase from *Zymomonas mobilis* (*Zm*PDC). *Zm*PDC is a tetramer, made up of a dimer of dimers, and there are four active sites per tetramer located at the interface between the subunits in each dimer [33]. One of the main reasons for using this enzyme for our study is that, unlike most other PDCs (e.g., from yeast), it does not show allosteric activation by its own substrate and thus gives normal Michaelis–Menten kinetics [34–38], and also it shows good stability. Previously the native enzyme expressed in *Escherichia coli* has been used in such studies but, in order to simplify the purification, in this study the gene was cloned into a pET28a vector, to give the enzyme an N-terminal His_6_-tag. This form of the protein was overexpressed in high yield in *E. coli*, and was easily purified on a Ni^2+^-NTA column. The His_6_-tag does not appear to affect the enzymic activity.

**Scheme 5:**
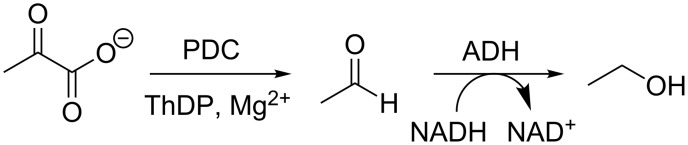
Coupled assay of PDC activity.

The normal coupled enzyme assay for PDC was used, in which PDC catalyses the decarboxylation of pyruvate to acetaldehyde and alcohol dehydrogenase (ADH) converts the acetaldehyde to ethanol using NADH (Scheme 5). The oxidation of NADH to NAD^+^ is monitored by UV spectroscopy at 340 nm.

**Figure 2:**
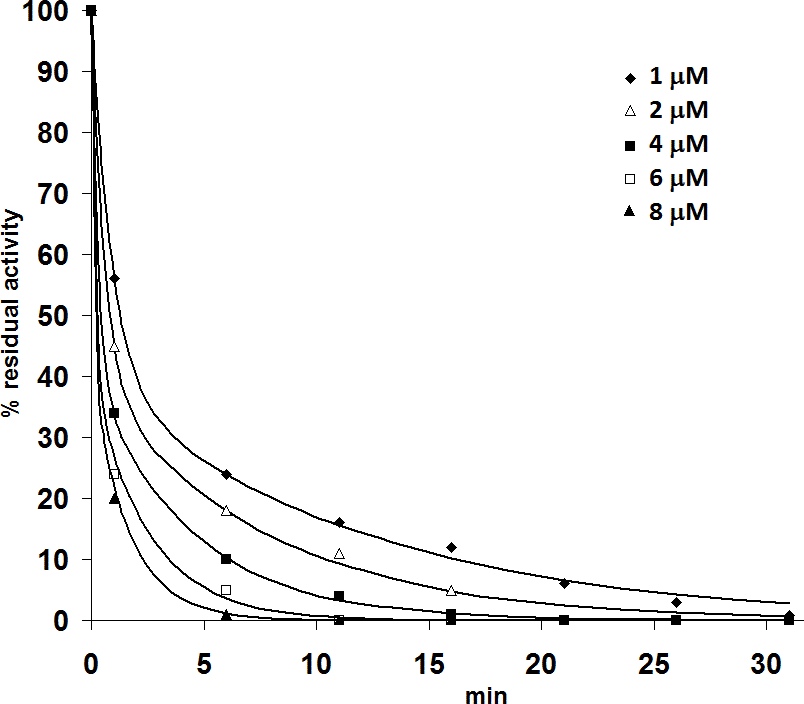
Time course inactivation of *Zm*PDC by various concentrations of furan **17**.

In order to follow the time-course of binding of furan **17**, various concentrations (1–8 µM) were incubated with apo-PDC in a Mg^2+^-containing buffer and aliquots were taken out at timed intervals (1–30 min) and added to the assay solution containing excess ThDP (100 µM). Under these conditions any apo-PDC that does not already have the furan analogue bound will bind ThDP and thus show activity. The plot of the percentage residual activity against time is shown in Figure 2. For all concentrations of inhibitor the enzyme lost all activity within 30 min, though the inhibition is faster at higher concentrations.

The data-points in Figure 2 fit poorly onto a simple exponential curve, however, a double exponential with 60% of the activity being lost at a fast rate and 40% being lost more slowly fits reasonably well; the equation used is


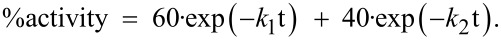


The faster of these two phases (*k*_1_) cannot be measured accurately from this data as it is almost all over within the first minute at all but the lowest concentrations of inhibitor. However, an estimated value of the second order rate constant *k*_on_ for the initial binding of **17** is ca. 1 µM^−1^ min^−1^.

The apparent two-stage inhibition for PDC was reported by Mann et al. for the benzene-based ThDP analogue **5** [10] and the same effect was also observed by Erixon et al. using triazole analogues **3** and **4** [37] and also with a thiazolone analogue by Kluger et al. [38]. It may be due to a slow conformational change after the initial binding or due to communication between the two active sites in each dimer.

**Figure 3:**
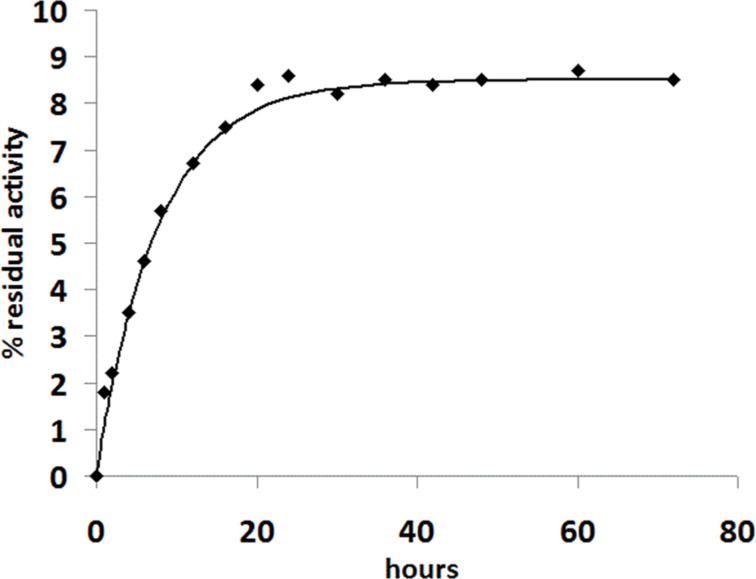
Recovery of activity for ZmPDC inhibited by furan **17** (1.0 µM) and then incubated with ThDP (1.0 mM). The activity is given as a percentage of the initial activity of uninhibited enzyme.

In order to measure a *K*_i_ value for furan **17** the enzyme has to be allowed to reach its equilibrium in a competition between binding ThDP and binding the analogue, and then the level of residual activity will indicate the proportion of active sites that have ThDP bound. Therefore, *Zm*PDC was inactivated by furan **17** (1 µM) and then a large excess of ThDP (1 mM) was added and the recovery in activity was followed over 3 days. Some activity was slowly recovered (Figure 3) but this reached a plateau within 24 hours at 8.5% of the untreated control (which remained fully active over this period). The steady activity regained in the presence of inhibitor is therefore the equilibrium position between ThDP binding and inhibitor binding.

The time-course for recovery of activity fits nicely to single exponential equation with an apparent first order rate constant of 0.13 h^−1^. Using the ratio of recovered activity over unrecovered activity at equilibrium (8.5:91.5), the relative concentrations of ThDP and inhibitor (1000:1) and the previously reported *K*_D_ value for ThDP (0.35 µM) [39], one can calculate that the *K*_i_ value of furan analogue **17** is 0.35 × (8.5/91.5) × (1/1000) µM = 32.5 pM. This *K*_i_ value is more than 10,000-fold lower than the *K*_D_ value for ThDP and is in the same range as previously reported for triazole analogues of ThDP (30 and 20 pM) [12,37]. However, the furan analogue of ThDP has the advantage over the triazole analogues that it can be functionalised at the C-2 position.

## Conclusion

In conclusion, we report here the short synthesis of a furan analogue of thiamine, which could be diphosphorylated to give an analogue of ThDP. Biological evaluation of this diphosphate showed that it is a very strong inhibitor of *Zm*PDC with picomolar affinity. Friedel–Crafts acetylation of the furan at C-2 was successful which opens the possibility of synthesising furan analogues of the enzymic reaction intermediates.

## Supporting Information

File 1 Experimental section along with ^1^H and ^13^C NMR spectra for all the compounds synthesised.
