# Supplementary material for: Gold(I)-catalysed synthesis of a furan analogue of thiamine pyrophosphate
Source: Beilstein J Org Chem. 2014 Nov 5;10:2580–5. doi: 10.3762/bjoc.10.270 (PMC4222410; doi:10.3762/bjoc.10.270)
Supplement: File 1 — Experimental section along with 1H and 13C NMR spectra for all the compounds synthesised. [file Beilstein_J_Org_Chem-10-2580-s001.pdf]

Supporting Information

for

Gold(I)-catalysed synthesis of a furan analogue of thiamine pyrophosphate

Amjid Iqbal, El-Habib Sahraoui and Finian J. Leeper*

Address: University of Cambridge, Department of Chemistry, Lensfield Road, Cambridge, CB2 1EW, U.K.

Email: Finian J. Leeper - fjl1@cam.ac.uk

*Corrsponding author

Experimental section along with ^{1}H and ^{13}C NMR spectra

for all the compounds synthesised

Experimental

General Synthesis Methods

All chemicals and reagents were obtained from Lancaster, Sigma-Aldrich and Acros. Silica gel used in flash chromatography was Merck Kieselgel 20 (230–400) mesh. Flash chromatography was carried out under a small positive pressure of air using distilled solvents. Analytical TLC was carried out on Merck glass plates with silica Kieselgel 60 F254 of thickness 0.25 mm and visualised under UV or $KMnO_4$ dip. All solvents were removed under reduced pressure using a Büchi rotary evaporator with dry ice traps. ^{1}H NMR was recorded using a Bruker AM/DPX 400 (400 MHz) or a Bruker DPX 500 (500

MHz) spectrometer and the number of protons attached determined by DEPT (Distortionless Enhancement by Polarisation Transfer) spectra. The chemical shifts are recorded in parts per million (ppm) downfield of TMS and coupling constants (*J*) are stated in Hz. ^{13}C NMR spectra were recorded on either a Bruker AM/DPX 400 (100 MHz) or a Bruker DPX 500 (126 MHz) spectrometer. Mass spectra used electron impact (EI) and electrospray ionisation (ESI). Melting points of compounds were measured using a Reichert machine and are uncorrected. IR spectra were obtained on a Perkin-Elmer FTIR spectrometer on neat samples.

*But-3-yn-1-yloxy(tert-butyl)dimethylsilane (**7**)*

To a stirred solution of but-3-ynol (3.0 mL, 40 mmol) in dry CH_2Cl_2 (20 mL) was added *N*-methylimidazole (9.52 mL, 120 mmol), iodine (0.8 g, 120 mmol) and *tert*-butyldimethylsilyl chloride (6.63 g, 44 mmol). After 4-5 h at room temperature, the solution was washed with saturated aq. $Na_2S_2O_3$, brine, and water, dried over $MgSO_4$ and evaporated under reduced pressure. Purification of the residue by column chromatography (hexane:EtOAc, 9:1, R_f 0.9) gave protected butynol **7** (7.1 g, 90%) as an oil. δ_H (400 MHz; $CDCl_3$) 0.06 (6H, s, *Me*$_2$Si), 0.89 (9H, s, ^{t}Bu), 1.94 (1H, t, *J* 2.4, C*H*≡C), 2.39 (2H, dt, *J* 2.4 and 7.2, OCH_2CH_2), 3.73 (2H, t, *J* 7.2, OC*H*$_2$); δ_C (100 MHz, $CDCl_3$) -5.31 *Me*$_2$Si), 18.3 (*C*Me$_3$), 22.8 (OCH_2CH_2), 25.8 (C*Me*$_3$), 61.7 (O*C*H$_2$), 69.2 (*C*H≡C), 81.5 (CH≡*C*); IR, ν_{max}/cm^{-1} 2857 (C-H), 2122 (C≡C), 1472 (C-H), 1103 (C-O).

*1,2-Bis(tert-butyldimethylsilyloxy)propan-2-one (**9**)*

To a stirred solution of dihydroxyacetone (dimer form, 1.8 g, 10 mmol) in dry THF (15 mL) was added *N*-methylimidazole (9.52 mL, 120 mmol), followed after 15 min by tert-butyldimethylsilyl chloride (6.63 g, 44 mmol) and iodine (0.8 g, 120 mmol). After 16 h, the solvent was evaporated. A solution of

the residue in CH_2Cl_2 was washed with saturated aq. $Na_2S_2O_3$, brine, and water and evaporated under reduced pressure. Purification of the residue by column chromatography (Hexane: EtOAc, 9.0: 1, R_f 0.7) gave the ketone **9** (7.0 g, 56%) as an oil. [Found: $M+Na^+$ (+ESI), 341.1929, $C_{15}H_{34}O_3Si_2$ requires M+Na, 341.1939]; δ_H (400 MHz; $CDCl_3$) 0.07 (12H, s, 2 x *Me*$_2$Si), 0.90 (18H, s, 2 x ^{t}Bu), 4.39 (4H, s, 2 x OC*H*$_2$); δ_C (100 MHz, $CDCl_3$) -5.55 (*Me*$_2$Si), 18.3 (Me$_3$*C*), 25.7 (*Me*$_3$C), 67.9 (O*C*H$_2$), 208.6 (*C*=O); IR, ν_{max}/cm^{-1} 2931 (C-H), 1731 (C=O), 1472 (C-H), 1253 (C-O).

*1,6-Bis(tert-butyldimethylsilyloxy)-2-(tert-butyldimethylsilyloxymethyl)hex-3-yn-2-ol (**10**)*

To a stirred solution of alkyne **7** (6.77 g, 36.7 mmol) in dry THF (20 mL) at -78 °C was added a solution of *n*-BuLi in hexane (36.71 mL, 40.43 mmol) dropwise. After 30 min, the solution was warmed to room temperature and stirred for a further 30 min and then was cooled again to -78 °C. A solution of ketone **9** (14.78 g, 36.76 mmol) in dry THF (20 mL) was added dropwise. The mixture was warmed to room temperature and stirred overnight. Saturated aq. NH_4Cl (20 mL) was then added and the mixture extracted with CH_2Cl_2. The combined extracts were washed with water and brine, dried over $MgSO_4$ and evaporated. Purification of the residue by column chromatography (Hexane:EtOAc, 9:1, R_f 0.56) gave the adduct **10** (2.75 g, 55%) as an oil [Found: $M+Na^+$ (+ESI), 525.3231, $C_{25}H_{54}O_4Si_3$ requires M+Na, 525.3222]; δ_H (400 MHz; $CDCl_3$) 0.04, 0.062 and 0.066 (each 6H, s, 3 x *Me*$_2$Si), 0.87 (9H, s, ^{t}Bu), 0.88 (18H, s, 2 x ^{t}Bu), 2.40 (2H, t, *J* 7.6, OCH$_2$C*H*$_2$), 3.57 and 3.67 (each 2H, d, *J* 9.5, 2 x C*H*$_2$O), 3.68 (2H, t, *J* 7.6, OC*H*$_2$CH$_2$); δ_C (100 MHz, $CDCl_3$) -5.35 (*Me*$_2$Si), 18.3 (Me$_3$*C*Si), 23.0 (OCH$_2$*C*H$_2$), 25.8 (*Me*$_3$C), 61.9 (O*C*H$_2$CH$_2$), 65.8 (2 x *C*H$_2$O), 71.1 (C≡C-*C*OH), 81.1 and 82.0 (*C*≡*C*); IR, ν_{max}/cm^{-1} 2929 (C-H), 2160 (C≡C), 1471 (C-H), 1100 (C-O).

*2-(Hydroxymethyl)hex-3-yne-1,2,6-triol (**11**)*

To a stirred solution of adduct **10** (7.57 g, 15 mmol) in dry THF (50 mL), was added TBAF (17.37 g, 60 mmol) portionwise. After 1 h the solvent was evaporated and the residue was purified by column chromatography (CH_2Cl_2:MeOH, 8:2, R_f 0.15) to give tetrol **11** (1.90 g, 79%) as an oil [Found: M+Na$^+$ (+ESI), 183.0628, $C_7H_{12}O_4$ requires M+Na, 183.0628]; δ_H (500 MHz; CD_3OD) 2.45 (2H, t, *J* 6.8, CH_2CH$_2$OH), 3.55 and 3.60 (each 2H, d, *J* 10.8, 2 x CH_2OH), 3.65 (2H, t, *J* 6.8, CH$_2$CH_2OH); δ_C (125 MHz, CD_3OD) 22.7 (*C*H$_2$CH$_2$OH), 60.6 (CH$_2$*C*H$_2$OH), 65.9 (2 x *C*H$_2$OH), 71.6 (C≡C-*C*OH), 81.3 and 83.1 (*C≡C*). IR, ν_{max}/cm^{-1} 3294 (O-H, broad), 2961 (C-H), 2150 (C≡C), 1461 (C-H), 1053 (C-O).

*2-(4-(Hydroxymethyl)furan-2-yl)ethanol (**12**)*

A solution of tetrol **11** (1.90 g, 11.87 mmol) in dry THF was added dropwise at 0 °C to an open flask containing a few crystals of AuCl. The mixture was stirred for 10 min and then filtered through a column of Celite. The filtrate was evaporated under reduced pressure to give furan **12** (1.66 g, 85%) in reasonably pure form. (EtOAc:MeOH, 7:3, R_f 0.2) [Found: M+Na$^+$ (+ESI), 165.0524, $C_7H_{10}O_3$ requires M+Na, 165.0522]; δ_H (400 MHz; CD_3OD) 2.78 (2H, t, *J* 6.8, CH_2CH$_2$OH), 3.76 (2H, t, *J* 6.8, CH$_2$CH_2OH), 4.40 (2H, s, ArCH_2OH), 6.13 and 7.31 (each 1H, s, ArH); δ_C (100 MHz, CD_3OD) 32.5 (*C*H$_2$CH$_2$OH), 56.7 (Ar*C*H$_2$OH), 61.2 (CH$_2$*C*H$_2$OH), 107.5 (Ar*C*H), 127.5 (Ar*C*), 139.5 (Ar*C*H), 155.1 (Ar*C*). IR, ν_{max}/cm^{-1} 3276 (OH, broad), 2961 (C-H), 1463 (C-H), 1025 (C-O).

The spectroscopic data for compounds **7** and **9**–**12** are consistent with those reported (subsequent to our work described above) by Deslongchamps and coworkers [1].

*5-(2-Hydroxyethyl)furan-3-carbaldehyde (**13**)*

A suspension of furan **12** (1.90 g, 13.37 mmol) and activated manganese oxide (11.62 g, 133.7 mmol) in chloroform (100 mL) was stirred at 25 °C for 8 h and then filtered. The filtrate was evaporated under reduced pressure and the residue was purified by silica gel chromatography (Hexane:EtOAc 7:3, R_f 0.43) to give aldehyde **13** (1.34 g, 72%) as an oil. [Found: M+Na$^+$ (+ESI), 163.0368, $C_7H_8O_3$ requires M+Na, 163.0366]; δ_H (400 MHz; $CDCl_3$) 2.91 (2H, t, *J* 6.3, C*H*$_2$CH$_2$OH), 3.90 (2H, t, *J* 6.3, CH$_2$C*H*$_2$OH), 6.50 and 7.96 (each 1H, s, ArH), 9.85 (1H, s, C*H*O); δ_C (100 MHz, $CDCl_3$) 31.2 (*C*H$_2$CH$_2$OH), 60.5 (CH$_2$*C*H$_2$OH), 103.5 (Ar*C*H), 129.5 (Ar*C*), 150.7 (Ar*C*H), 156.1 (Ar*C*), 184.6 (*C*HO); IR, ν_{max}/cm^{-1} 3402 (OH, broad), 3126 (=C-H), 2927 (C-H), 1671 (C=O), 1543 (C-H), 1041 (C-O).

*3-(5-(2-Hydroxyethyl)furan-3-yl)-2-(phenylaminomethyl)acrylonitrile (**14**)*

A mixture of aldehyde **13** (820 mg, 5.85 mmol) and β-anilinopropionitrile (1.02 g, 7.02 mmol) in dry dimethyl sulfoxide (20 mL) was heated to 45 °C under argon. A solution of NaOMe, prepared by dissolving Na metal (94.7 mg, 1.75 mmol) in methanol (3 mL) was added dropwise. The mixture was stirred at 45 °C for 3 h, then poured into ice-water (30 mL) and extracted with CH_2Cl_2. The combined organic layers were washed with brine, dried over $MgSO_4$ and evaporated. Silica gel chromatography (Hexane:EtOAc, 7:3, R_f 0.6) gave the acrylonitrile **14** (1.2 g, 76%) as a solid, which seemed by NMR to be predominantly one double bond isomer. [Found: M+H$^+$ (+ESI), 269.1294, $C_{16}H_{17}N_2O_2$ requires M+H, 269.1285]; δ_H (400 MHz; $CDCl_3$) 1.70 (2H, br, OH and NH), 2.92 (2H, t, *J* 6.4, C*H*$_2$CH$_2$OH), 3.89 (2H, t, *J* 6.4, CH$_2$C*H*$_2$OH), 4.08 (2H, s, CH$_2$), 6.67 (2H, d, *J* 8, phenyl o-*H*), 6.82 (2H, t, *J* 8, phenyl p-*H*), 6.88 (1H, s, CH), 7.01 (1H, s, CH), 7.23 (2H, t, *J* 8, phenyl m-*H*), 7.66 (1H, s, CH); δ_C (100 MHz, $CDCl_3$) 31.9 (*C*H$_2$CH$_2$OH), 47.7 (CH$_2$), 61.1 (CH$_2$*C*H$_2$OH), 105.6 (Ar*C*H), 113.5 (phenyl o-*C*), 115.5 and 115.7 (CN and C=*C*) 118.9 (phenyl p-*C*), 129.8 (phenyl m-*C*), 130.2 (Ar*C*), 134.3 (Ar*C*H), 144.8 (C=*C*H), 155.5

(Ar*C*), 184.6 (*C*HO); IR, ν_{max}/cm^{-1} 3373 (OH), 3026 (N-H), 3016 (Ph C-H), 2933 (C-H), 2247 (CN), 1690 (C=C), 1602 (Ph C=C), 1499 (Ph C=C), 1136 (C-O).

*2-(4-(4-Amino-2-methylpyrimidin-5-ylmethyl)furan-2-yl)ethanol (**15**)*

To a stirred solution of acrylonitrile **14** (1.2 g, 4.47 mmol) in dry ethanol (30 mL) was added acetamidine hydrochloride (568 mg, 6.04 mmol) and dropwise a solution of NaOEt, prepared by dissolving Na metal (411.24 mg, 17.88 mmol) in dry ethanol (5 mL). The mixture was heated at reflux for 48 h, then evaporated under reduced pressure. Purification of the residue by column chromatography (CH_2Cl_2:MeOH, 9.5:0.5, R_f 0.2) gave the pyrimidine **15** (678.0 mg, 65%) as a solid [Found: $M+H^+$ (+ESI), 234.1232, $C_{12}H_{15}N_3O_2$ requires M+H, 234.1237]; δ_H (400 MHz; CD_3OD) 2.41 (3H, s, pyrimidine-C*H₃*), 2.78 (2H, t, *J* 7.2, C$H_2$$CH_2$OH), 3.53 (2H, s, ArC$H_2$Ar), 3.75 (2H, t, *J* 7.2, CH_2OH), 6.01 (1H, s, furan 3-H), 7.24 (1H, s, furan 5-H), 7.81 (1H, s, pyrimidine-*H*); δ_C (100 MHz, CD_3OD, HMQC) 23.8 (pyrimidine-*C*H_3), 24.2 (Ar*C*H_2Ar), 32.6 (*C*H_2CH_2OH), 61.2 (CH_2*C*H_2OH), 108.5 (furan C-3), 115.2 (*C*=CNH_2), 122.6 (furan C-4), 140.0 (furan C-5), 151.1 (pyrimidine *C*H), 155.6 (furan C-2), 164.1 and 165.0 (*C*N*C*NH_2); ν_{max}/cm^{-1} 3331 (OH), 3167 (NH_2), 2919, 1661 (NH_2), 1599, 1555 (pyrimidine ring), 1031 (C-O).

*2-(4-(4-Amino-2-methylpyrimidin-5-ylmethyl)furan-2-yl)ethyl tosylate (**16**)*

To a solution of alcohol **15** (80.0 mg, 0.34 mmol) in pyridine (2 mL) at -5 °C was added portionwise *p*-toluenesulfonyl chloride (324.0 mg, 1.7 mmol). The mixture was stirred at -5 °C for 3 h, then treated with cold hydrochloric acid (1 M, 2 mL), neutralised with $NaHCO_3$ and extracted with CH_2Cl_2 (3 x 10 mL). The combined organic layers were washed with saturated aq. $CuSO_4$ and brine, dried over $MgSO_4$ and evaporated under reduced pressure. Purification of the residue by silica gel

chromatography (CH_2Cl_2:MeOH 9.5:0.5, R_f 0.3) gave the tosylate **16** (96 mg, 72%) as a solid [Found: M + H^+ (+ESI), 388.1308, $C_{19}H_{21}N_3O_4S$ requires M + H, 388.1326]; δ_H (400 MHz; $CDCl_3$) 2.42 and 2.53 (each 3H, s, C*H*$_3$), 2.91 (2H, t, *J* 6.6, C*H*$_2$CH$_2$O), 3.52 (2H, s, ArC*H*$_2$Ar), 4.18 (2H, t, *J* 6.6, CH$_2$C*H*$_2$O), 5.68 (2H, br s, N*H*$_2$), 5.89 (1H, s, furan 3-H), 7.11 (1H, s, furan 5-H), 7.31 and 7.72 (each 2H, d, *J* 8.1, tosyl-*H*), 7.92 (1H, s, pyrimidine-*H*); δ_C (100 MHz, $CDCl_3$) 21.6, 24.0 (2 x *C*H$_3$), 24.1 and 28.1 (2 x *C*H$_2$), 67.5 (*C*H$_2$O), 108.3 (furan C-3), 112.8 (*C*=CNH$_2$), 120.8 (furan C-4), 127.8, 129.9 (2 × tosyl *C*H), 132.7 (tosyl C), 139.1 (furan C-5), 145.0 (tosyl C), 150.7 (br, pyrimidine *C*H), 151.7 (furan C-2), 162.1, 164.4 (*C*N*C*NH$_2$); ν_{max}/cm^{-1} 3392 (NH_2), 1601, 1498 (pyrimidine ring), 1399 (SO_2O), 1203 (aromatic C-H).

*2-(4-(4-Amino-2-methylpyrimidin-5-ylmethyl)furan-2-yl)-ethyl diphosphate (**17**)*

To a stirred solution of tosylate **16** (41.0 mg, 0.1 mmol) in anhydrous acetonitrile (1 ml) at 4 °C under an atmosphere of argon was added in portions tris(tetra-*n*-butylammonium) hydrogen diphosphate (181.0 mg, 0.2 mmol). The mixture was stirred at 4 °C for 12 h under nitrogen, then diluted with water (1 mL) and purified by HPLC. Lyophilisation gave diphosphate **17** as a white powder (12.4 mg, 30%).[Found: M+H^+ (+ESI), 394.0563, $C_{12}H_{17}N_3O_8P_2$ requires M+H, 394.0564]; δ_H (500 MHz, D_2O) 2.31 (3H, s, pyrimidine-C*H*$_3$), 2.79 (2H, t, *J* 6.5, C*H*$_2$CH$_2$O), 3.43 (2H, s, ArCH$_2$Ar), 3.97 (2H, q, *J* 6.5, CH$_2$C*H*$_2$O), 6.03 (1H, s, furan 3-H), 7.13 (1H, s, furan 5-H), 7.66 (1H, s, pyrimidine-C*H*); δ_C (125 MHz; D_2O) 21.6 (*C*H$_3$), 22.7 and 28.8 (2 x *C*H$_2$), 63.5 (*C*H$_2$O), 107.7 (furan C-3), 114.7 (*C*=CNH$_2$), 120.4 (furan C-4), 139.1 (furan C-5), 154.0 (furan C-2); δ_P (162 MHz, D_2O) -7.15 and -10.12 (each 1P, d, *J* 19.5).

*2-(5-Acetyl-4-(4-amino-2-methylpyrimidin-5-ylmethyl)furan-2-yl)ethyl acetate (**18**)*

To a stirred solution of alcohol **15** (120 mg, 0.51 mmol) in CH_2Cl_2 (5 mL) at 0 °C under an atmosphere of argon was added aluminium trichloride (470.0 mg, 3.52 mmol) and acetyl chloride (0.46 g, 5.87

mmol). The mixture was allowed to warm to room temperature, stirred for 3 h, then poured into ice-water (10 mL), neutralised with $NaHCO_3$ and extracted with CH_2Cl_2 (3 x 10 mL). The combined organic layers were washed with brine, dried over $MgSO_4$ and evaporated. Purification of the residue by column chromatography (CH_2Cl_2:MeOH 9.5:0.5, R_f 0.13) gave the acetate **18** (117.1 mg, 67%) as a solid [Found: $M+H^+$ (+ESI), 318.1442, $C_{16}H_{19}N_3O_4$ requires M+H, 318.1448]; δ_H (400 MHz; CD_3OD) 1.97 (3H, s, OAc), 2.37 (3H, s, pyrimidine-CH_3), 2.48 (3H, s, 5-$COCH_3$), 3.01 (2H, t, *J* 6.5 Hz, CH_2CH_2O), 3.91 (2H, s, ArCH_2Ar), 4.33 (2H, t, *J* 6.5 Hz, CH_2O), 6.32 (1H, s, furan 3-H), 7.94 (1H, s, pyrimidine-*H*); δ_C (100 MHz; CD_3OD, HMBC) 20.6 ($OCOCH_3$), 24.5 (pyrimidine-CH_3), 25.1 (ArCH_2Ar), 26.8 (5-$COCH_3$), 28.8 (CH_2CH_2O), 62.6 (CH_2O), 113.3 (furan C-3), 113.3 ($C{=}CNH_2$), 133.5 (furan C-4), 148.8 (furan C-5), 154.2 (pyrimidine-*C*H), 158.2 (furan C-2), 163.5 ($CNCNH_2$), 166.4 ($CNCNH_2$), 172.4 (*C*OO), 191.8 (*C*O); ν_{max}/cm^{-1} 3405, 3330 (NH_2), 1739 (COO), 1666 (C=O), 1657 (NH_2), 1601, 1561 (pyrimidine ring).

Cloning of the gene for ZmPDC into pET28a

An *E. coli* strain having the gene for *Zm*PDC present in pPLZM plasmid [2] was incubated overnight at 37 °C with shaking at 250 rpm in LB liquid medium supplemented with 100 µg/mL carbenicillin. The plasmid was purified using Plasmid Miniprep Kit (Fermentas) and checked for purity and approximate plasmid size using agarose gel electrophoresis. The gene was then amplified by PCR using primers PDCf and PDCr, designed for subsequent assembly with pET28a by isothermal assembly [3].

PDCf: 5’-GGTGCCGCGCGGCAGCCATATGAGTTATACTGTCGGTACCTATTTAGGG-3’ and

PDCr: 5’-TGGTGCTCGAG TGCGGCCGCCTAGAGGAGCTTGTTAACAGGCTTACGGCT-3’.

PCR involved: initial denaturation at 95 °C for 3 min, then 25 cycles of 30 sec at 95 °C, annealing at 55 °C for 30 sec and extension at 72 °C for 3 min, and a final extension time of 10 min at 72 °C. The PCR reaction mixture contained: 1X KOD buffer, 2 mM $MgSO_4$, 0.2 mM dNTP, 6% DMSO, 1 µl PDC

plasmid, 1 µL of each primer (50 mM), 1 µL KOD polymerase and made up with autoclaved deionised MQ water to 50 µL.

The PCR product was checked and purified by agarose gel electrophoresis [Figure S1(B)]. pET-28a was digested with restriction enzymes *NheI* and *NotI* and purified by agarose gel electrophoresis using Gel Extraction Kit (Fermentas). The purified PDC PCR fragment and the digested pET-28a were then added to an isothermal assembly reaction mixture [3]. After incubation at 50 °C for 2 h, 2 µL was used to transform chemically competent NovaBlue *E. coli* using standard heat-shock method before plating on LB-plates supplemented with 50 µg/mL kanamycin. Overnight incubation at 37 °C resulted in tens of colonies, of which few were selected and grown in liquid LB-medium. The plasmids of the selected colonies were isolated and checked for size using agarose gel electrophoresis. Sequencing using T7 and T7t sequencing primers confirmed that the desired plasmid had been acquired. This plasmid was then used to transform chemically competent *E.coli* BL-21 cells using the heat-shock method. The latter gave tens of colonies, of which one was selected and grown in liquid medium supplemented with 50 µg/mL kanamycin and a stock was made in 20% glycerol and stored at -80 °C.

Overexpression and purification of His_6-tagged PDC

Frozen stock was used to inoculate an overnight 10 mL LB starter culture. 1 mL of the starter culture was then used to inoculate 1 litre of LB supplemented with 50 µg/mL kanamycin and incubated at 37 °C with continuous shaking at 250 rpm. Protein expression was induced by the addition of 200 µM IPTG when OD_{600} reached ~0.8, and the shaking and temperature were reduced to 200 rpm and 16 °C respectively.

All the following manipulations were carried out at 4 °C. Cells were pelleted by centrifugation at 7,000 rpm (12,227 G) for 10 min. The cell pellet was then resuspended in 30 mL of binding buffer (20 mM Tris-HCl, pH 7.9, 0.5 M NaCl, 5 mM imidazole, 10% glycerol). The resuspended cells were then

lysed by sonication (2 sec on and 10 sec off, 120 cycles). The lysate was then centrifuged at 17,000 rpm (34,957 G) for 40 min and the supernatant was loaded to a column containing 1 mL His•Bind™ resin (Novagen) that had been washed with 10 mL of sterile MQ H_2O, charged with 10 mL of 50 mM $NiCl_2$ and pre-equilibrated 15 mL of binding buffer. The bound protein was washed with 10 mL binding buffer (5 mM imidazole) and 5 mL wash buffer (20 mM Tris-HCl, pH 7.9, 0.5 M NaCl, 60 mM imidazole, 10% glycerol) prior to elution with 4 mL elution buffer (20 mM Tris-HCl, pH 7.9, 0.5 M NaCl, 200 mM imidazole, 20% glycerol). The eluate was concentrated and buffer exchanged by repeated ultrafiltration (cut off 30 KDa) and stored in 50 mM MES buffer pH 6.8 containing 0.1 mM TPP, 2 mM $MgCl_2$, 1 mM DTT and 50% glycerol and kept at -20 °C. A protein sample was run on sodium dodecylsulfate/polyacrylamide gel electrophoresis (SDS-PAGE) gel to confirm size and purity [4]. The gel showed a single band of ~70 KDa which corresponded to the monomer (theoretical mass: 62,958 Da including His-tag but without the first methionine). The protein yield, estimated using Bradford assay [5], was *ca.* 10 mg per litre.

Enzyme assays and inhibition experiments

The assay of PDC activity, preparation of apo-enzyme, incubation with ThDP analogue **17** [6] and partial reactivation of the enzyme using excess ThDP [7] were carried out as previously described.

Supplementary Material - NMR Spectra

Tris-(TBDMS) ether **10** in $CDCl_3$

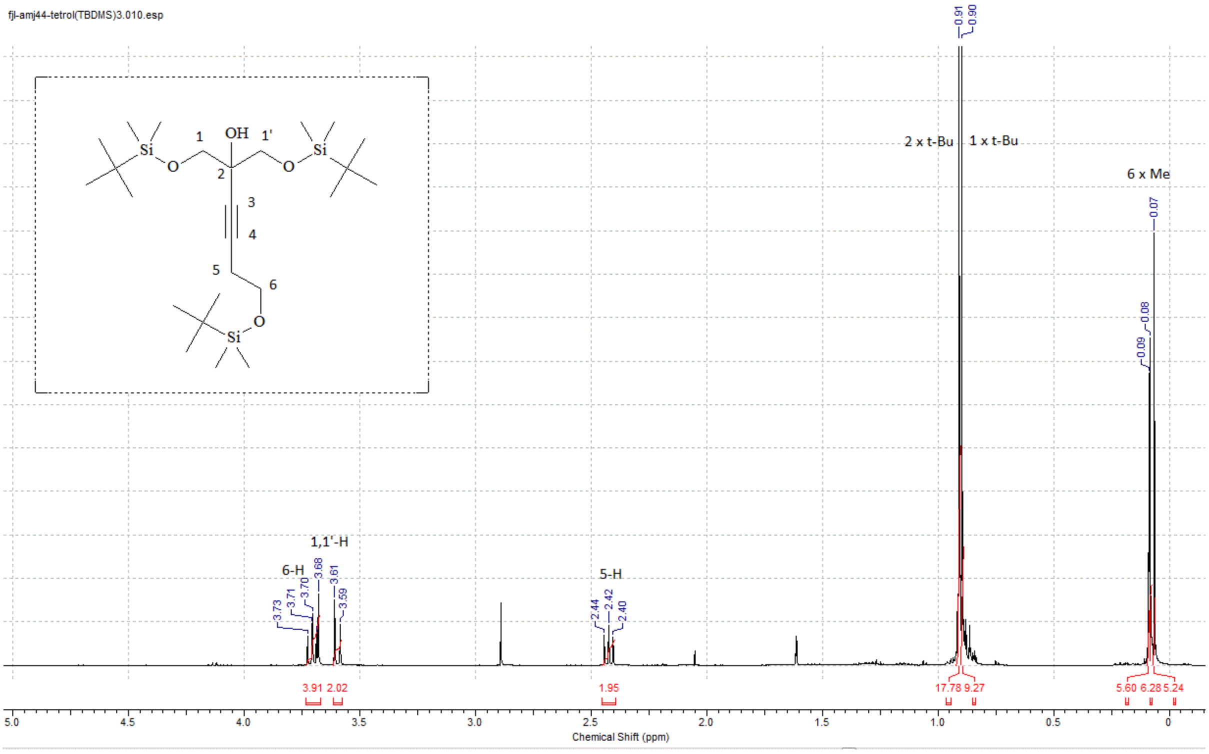

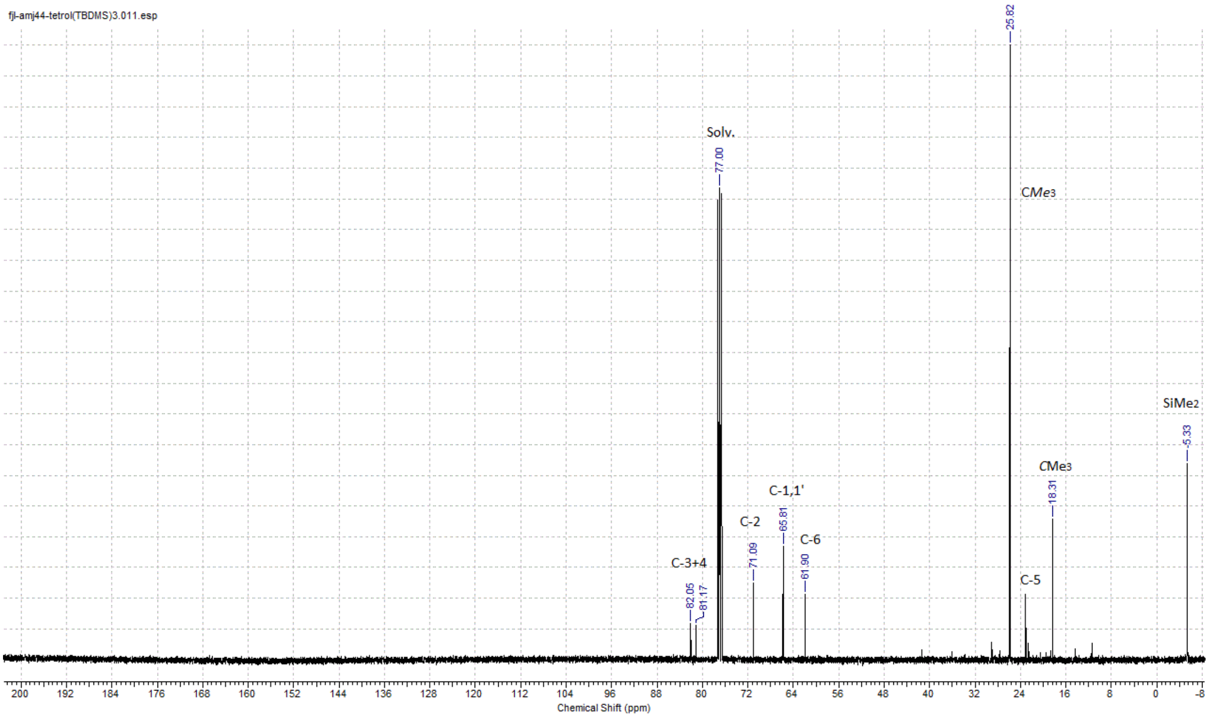

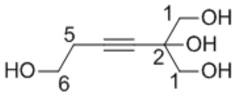

1
OH
5
OH
2
HO
6
1
OH

11 in CD_3OD

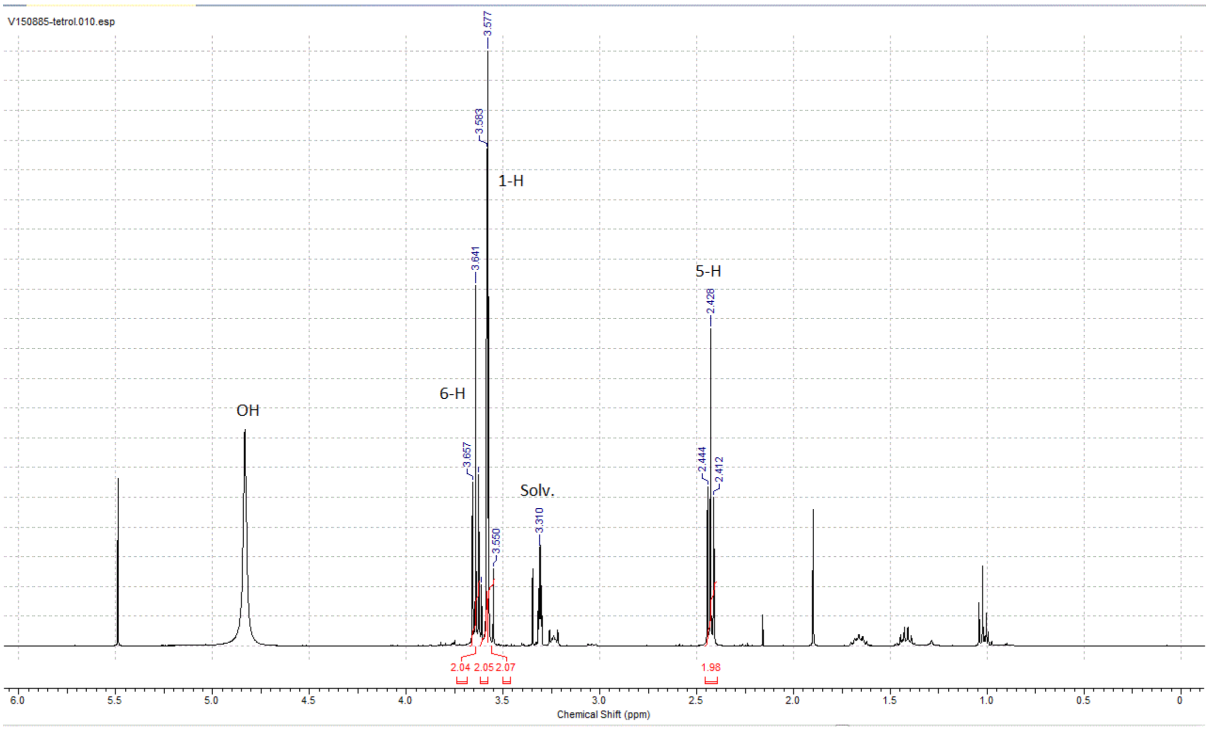

V150885-tetrol.010.esp
3.577
3.583
1-H
3.641
5-H
2.428
6-H
OH
3.657
2.444
2.412
Solv.
3.310
3.550
2.04
2.05
2.07
1.98
6.0
5.5
5.0
4.5
4.0
3.5
3.0
2.5
2.0
1.5
1.0
0.5
0
Chemical Shift (ppm)

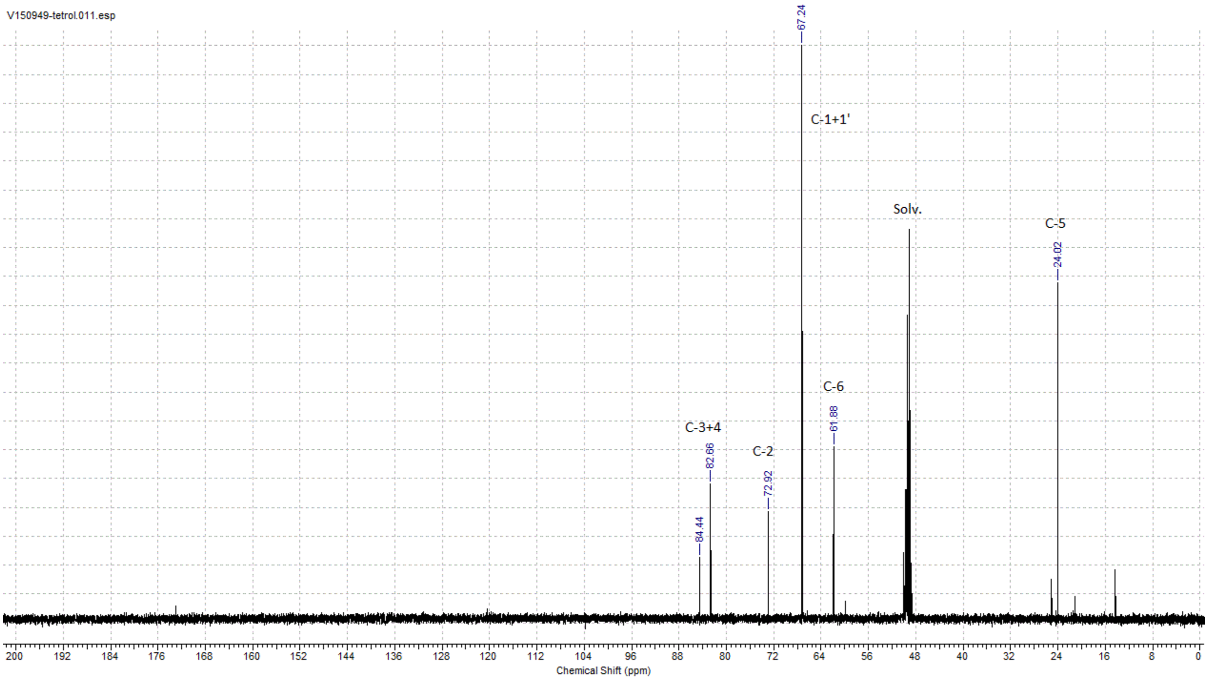

V150949-tetrol.011.esp
67.24
C-1+1'
Solv.
C-5
24.02
C-6
61.88
C-3+4
82.66
C-2
72.92
84.44
200
192
184
176
168
160
152
144
136
128
120
112
104
96
88
80
72
64
56
48
40
32
24
16
8
0
Chemical Shift (ppm)

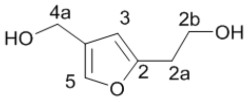
HO
4a
3
2b
OH
5
O
2
2a

12 in CD_3OD

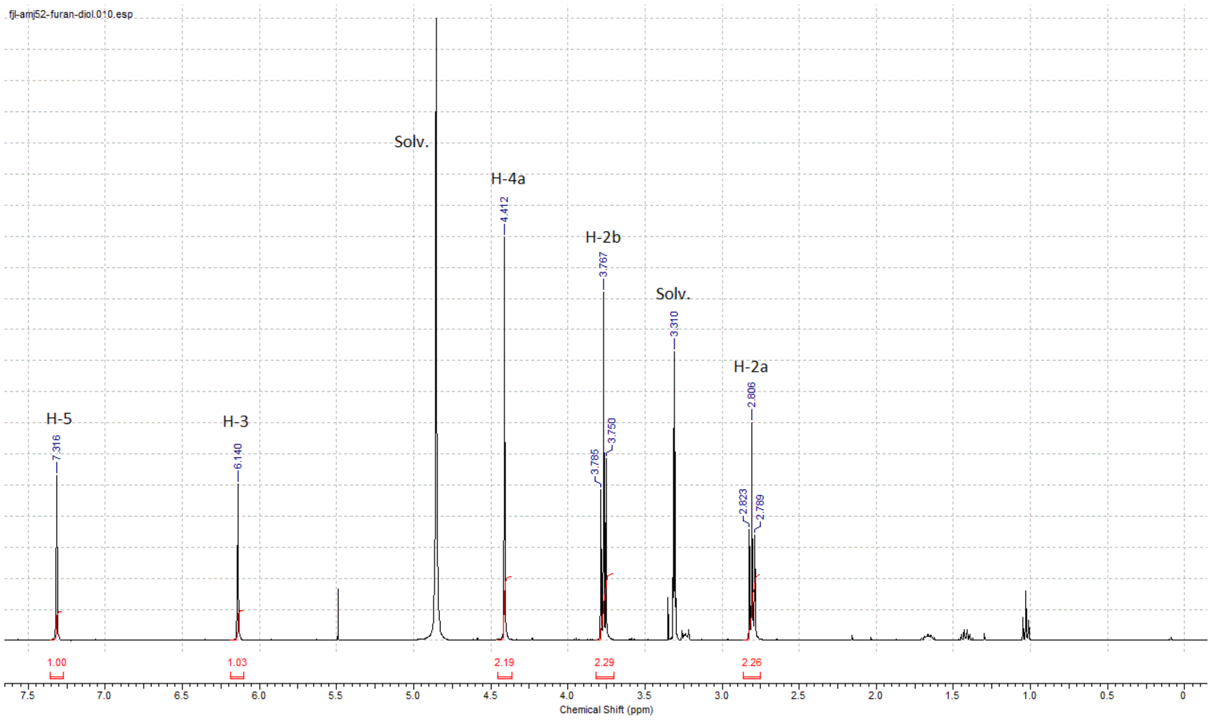
fjl-amj52-furan-diol.010.esp
Solv.
H-4a
4.412
H-2b
3.767
Solv.
3.310
H-2a
2.806
H-5
7.316
H-3
6.140
3.785
3.750
2.823
2.789
1.00
1.03
2.19
2.29
2.26
Chemical Shift (ppm)

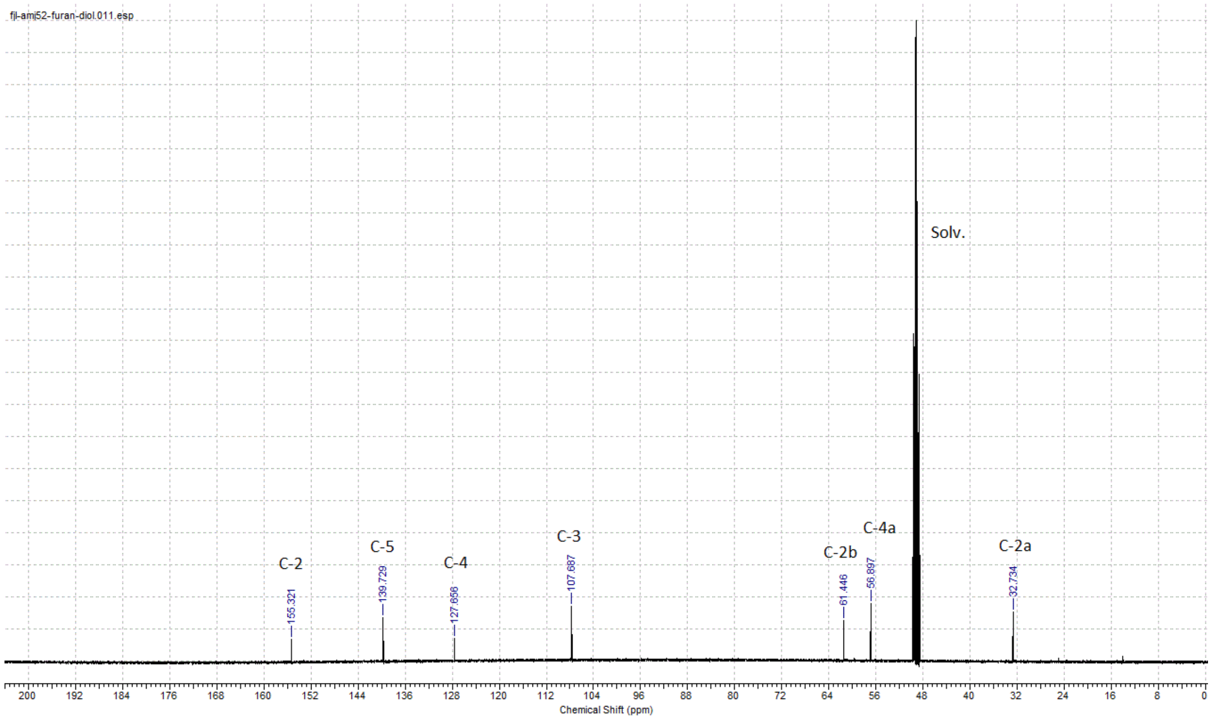
fjl-amj52-furan-diol.011.esp
Solv.
C-4a
C-2b
C-2a
C-3
C-5
C-4
C-2
155.321
139.729
127.656
107.687
61.446
56.897
32.734
Chemical Shift (ppm)

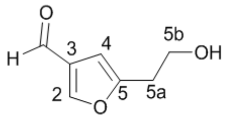
O
H
3
4
5b
OH
2
5
5a
O

13 in $CDCl_3$

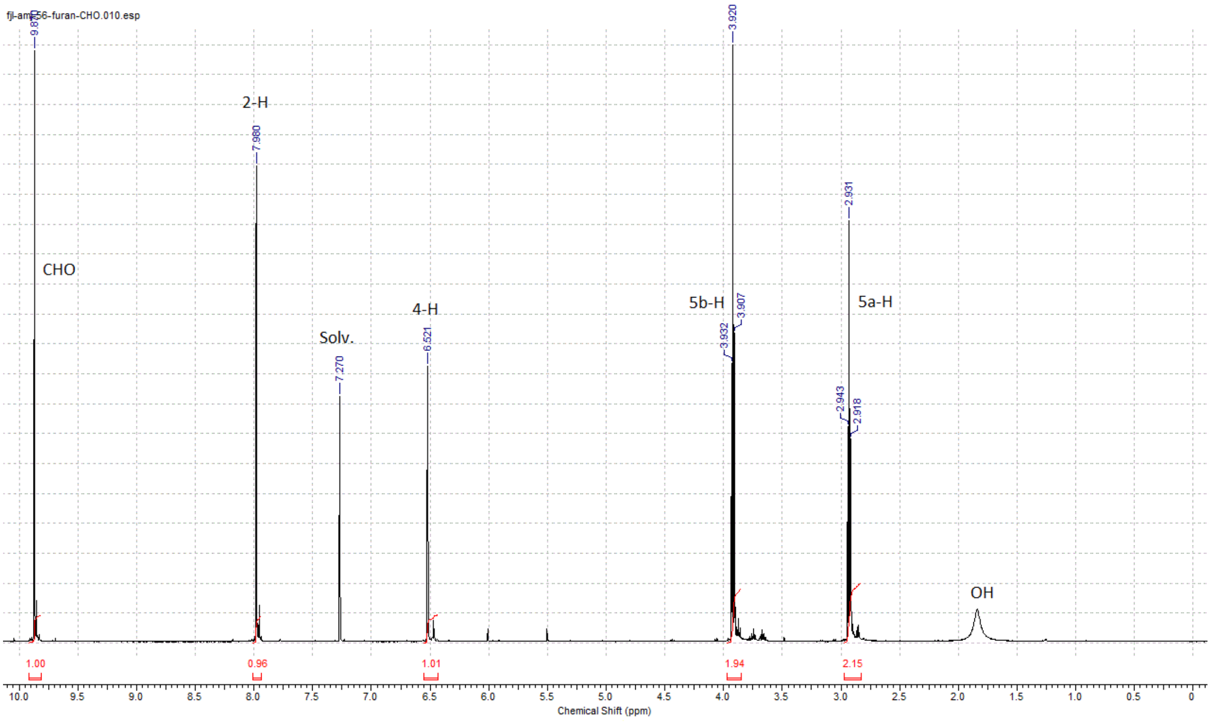
fjl-ami-56-furan-CHO.010.esp
CHO
2-H
Solv.
4-H
5b-H
5a-H
OH
7.980
7.270
6.521
3.920
3.932
3.907
2.931
2.943
2.918
1.00
0.96
1.01
1.94
2.15
10.0 9.5 9.0 8.5 8.0 7.5 7.0 6.5 6.0 5.5 5.0 4.5 4.0 3.5 3.0 2.5 2.0 1.5 1.0 0.5 0
Chemical Shift (ppm)

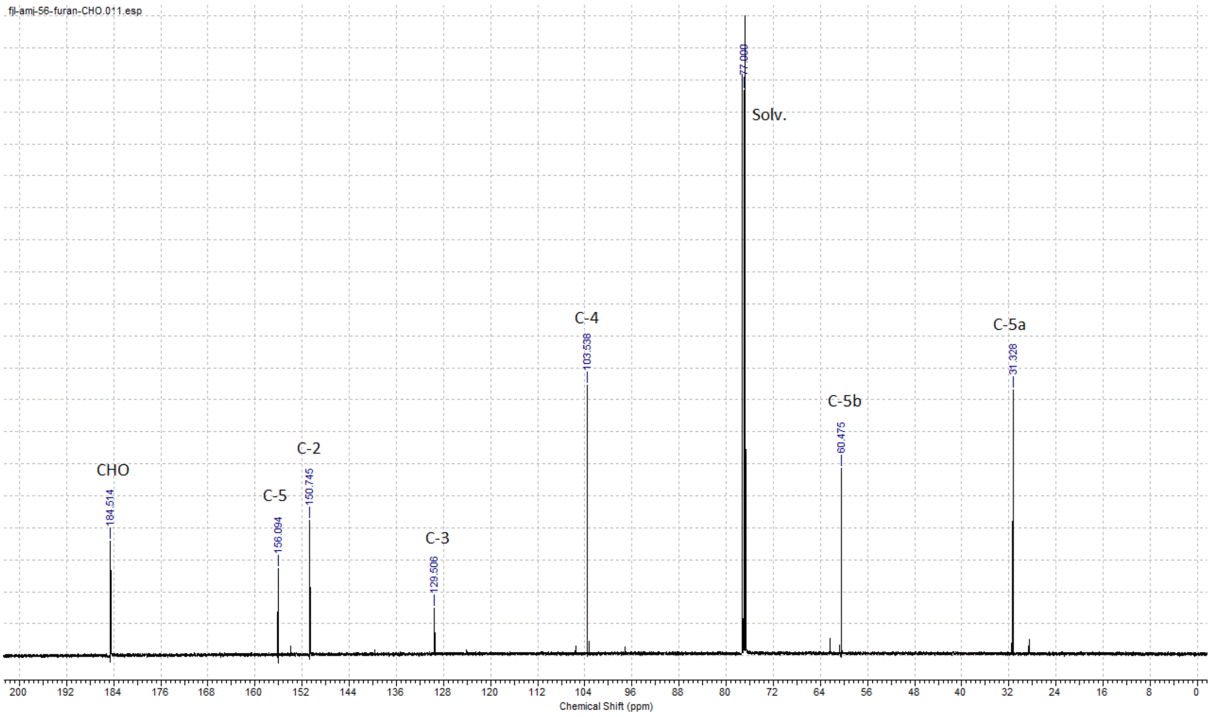
fjl-ami-56-furan-CHO.011.esp
Solv.
77.000
C-4
103.538
C-5a
31.328
C-5b
60.475
C-2
150.745
CHO
184.514
C-5
156.094
C-3
129.506
200 192 184 176 168 160 152 144 136 128 120 112 104 96 88 80 72 64 56 48 40 32 24 16 8 0
Chemical Shift (ppm)

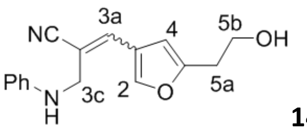

NC
3a
4
5b
OH
Ph
N
H
3c
2
O
5a

14 in $CDCl_3$

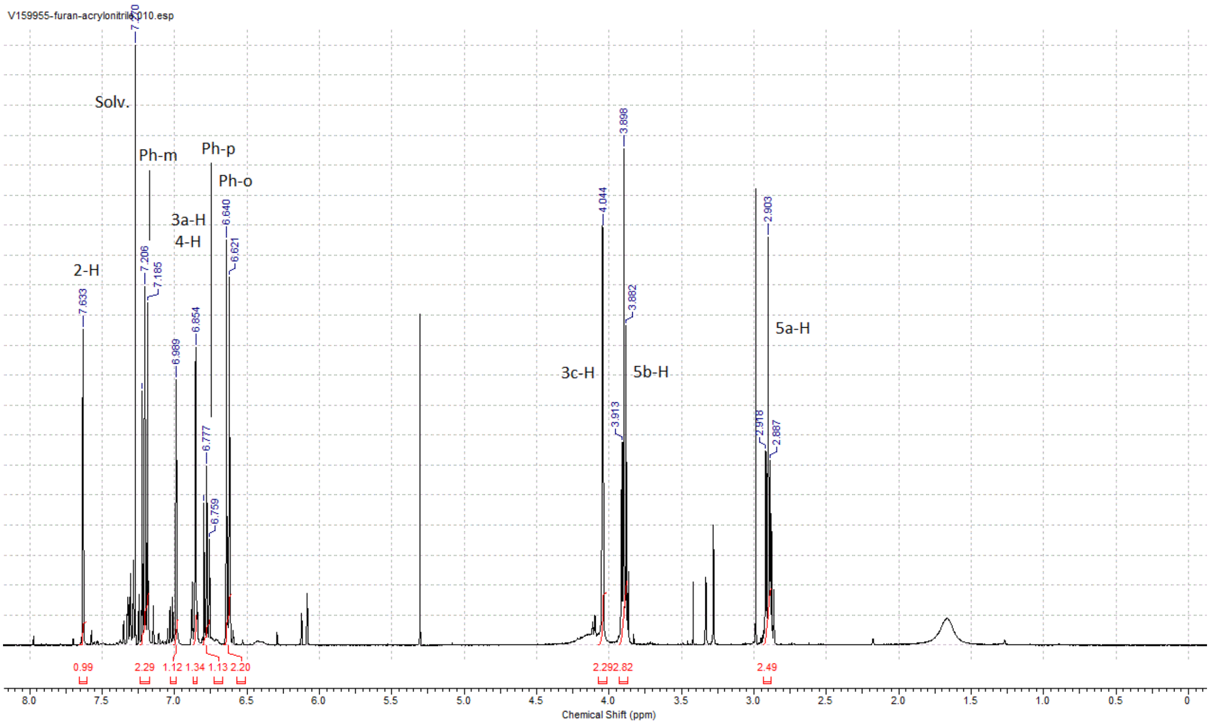

V159955-furan-acrylonitrile.010.esp
Solv.
Ph-m
Ph-p
Ph-o
3a-H
4-H
2-H
3c-H
5b-H
5a-H
7.633
7.206
7.185
6.989
6.854
6.777
6.759
6.640
6.621
4.044
3.913
3.898
3.882
2.918
2.903
2.887
0.99
2.29
1.12
1.34
1.13
2.20
2.29
2.82
2.49
Chemical Shift (ppm)

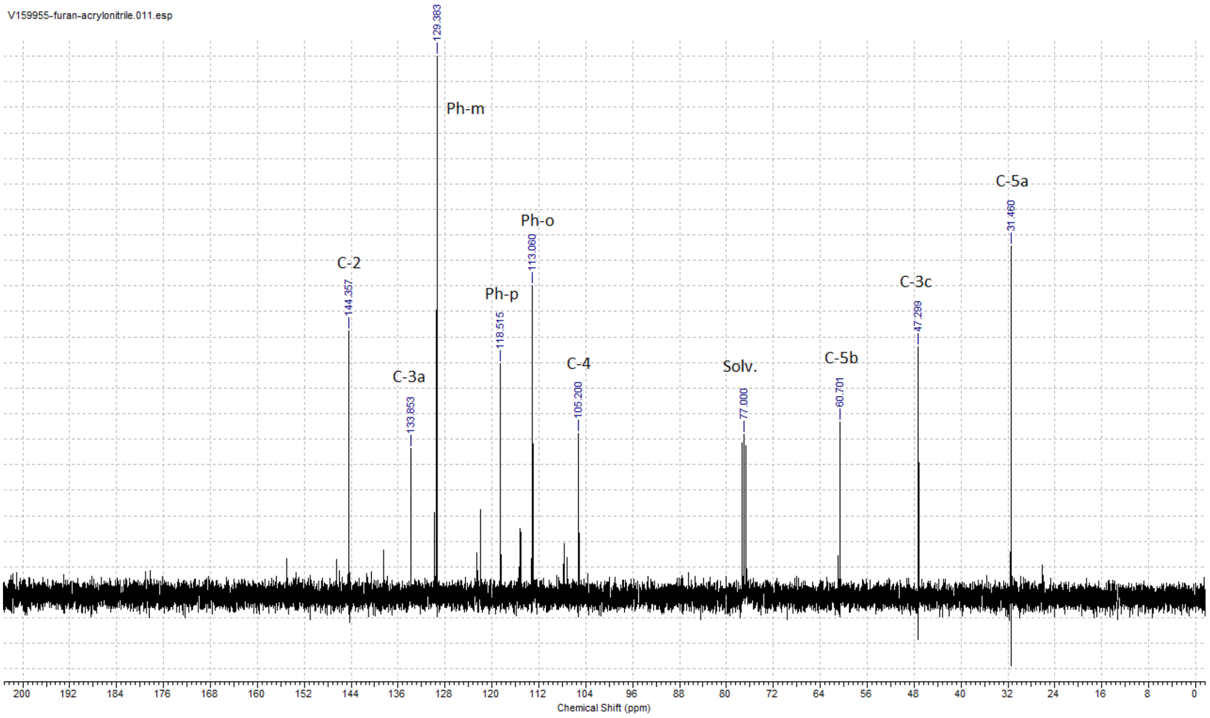

V159955-furan-acrylonitrile.011.esp
129.383
Ph-m
C-5a
31.460
Ph-o
113.060
C-2
144.357
Ph-p
118.515
C-3c
47.299
C-3a
133.853
C-4
105.200
Solv.
77.000
C-5b
60.701
Chemical Shift (ppm)

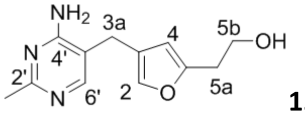

15 in CD_3OD

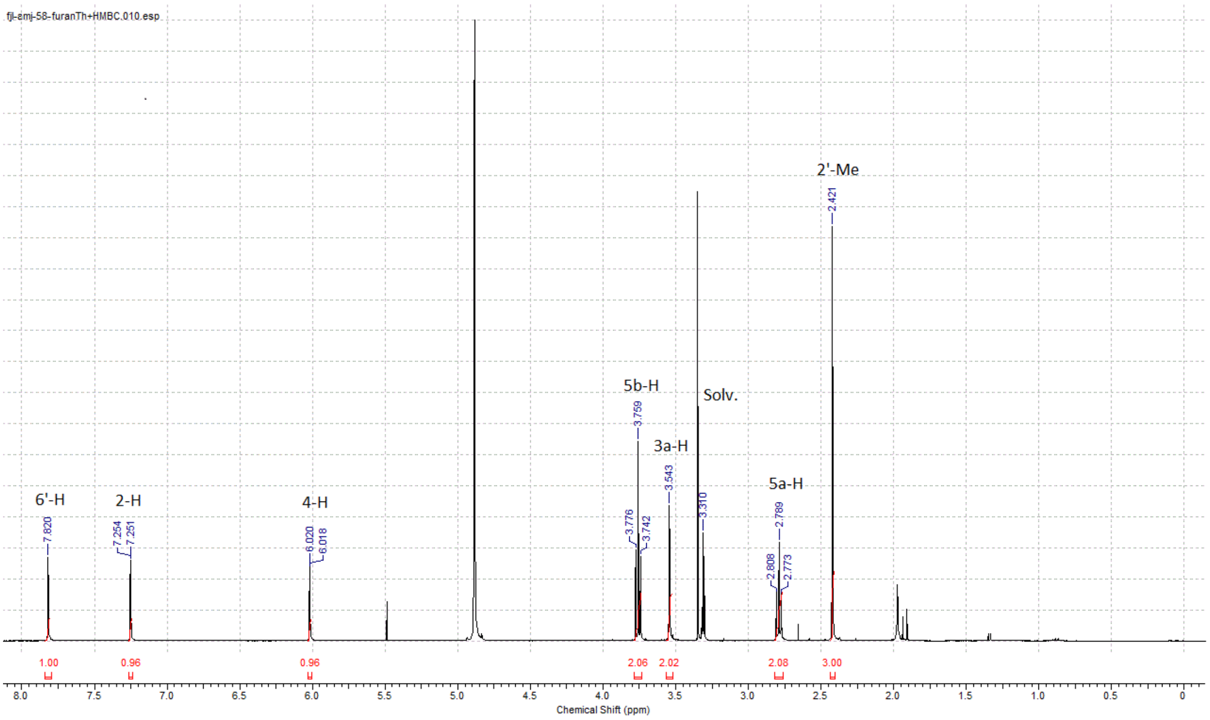
fjl-amj-58-furanTh+HMBC.010.esp
6'-H
2-H
4-H
5b-H
3a-H
Solv.
5a-H
2'-Me
7.820
7.254
7.251
6.020
6.018
3.776
3.759
3.742
3.543
3.310
2.808
2.789
2.773
2.421
1.00
0.96
0.96
2.06
2.02
2.08
3.00
Chemical Shift (ppm)

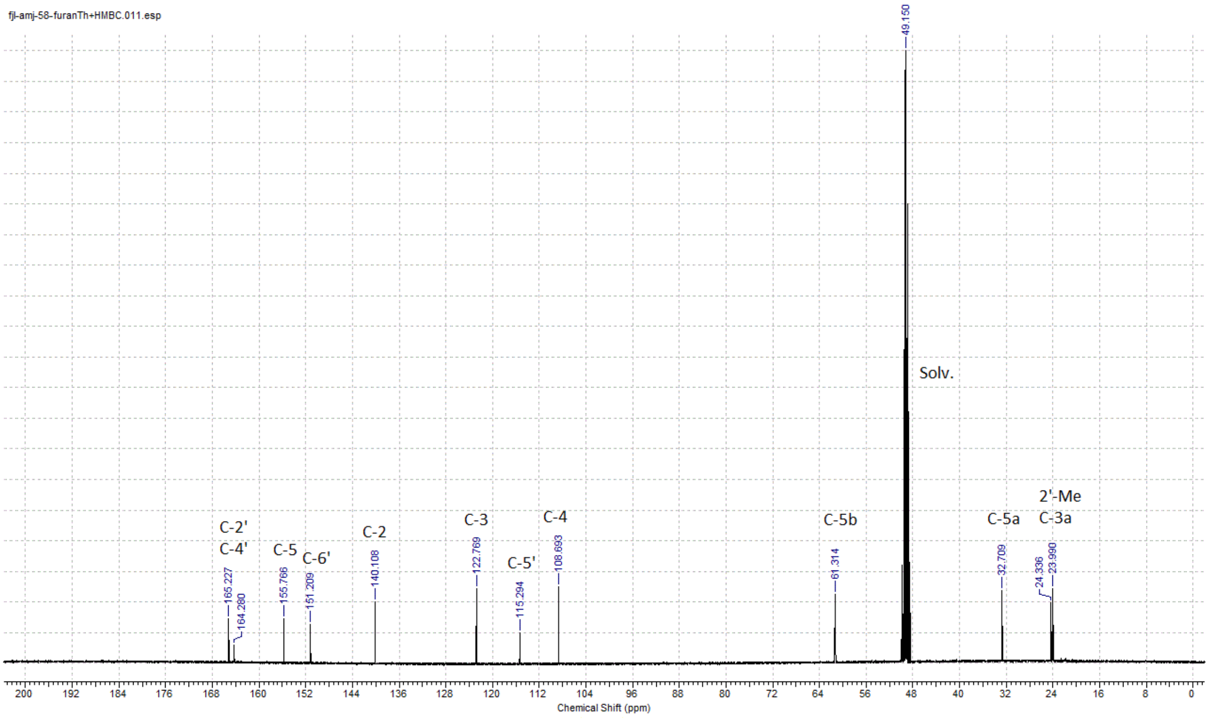
fjl-amj-58-furanTh+HMBC.011.esp
C-2'
C-4'
C-5
C-6'
C-2
C-3
C-5'
C-4
C-5b
Solv.
C-5a
2'-Me
C-3a
165.227
164.280
155.766
151.209
140.108
122.769
115.294
108.693
61.314
49.150
32.709
24.336
23.990
Chemical Shift (ppm)

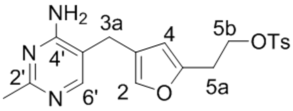
NH2
3a
4
5b
OTs
N
4'
2'
N
6'
2
O
5a

16 in $CDCl_3$

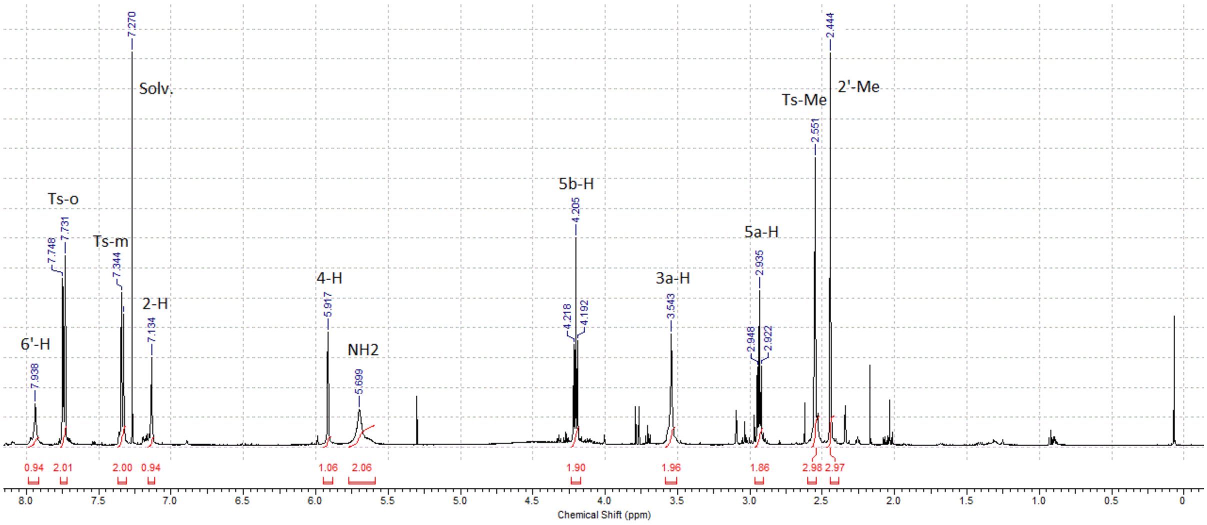
Solv.
7.270
Ts-o
7.748
7.731
Ts-m
7.344
2-H
7.134
6'-H
7.938
4-H
5.917
NH2
5.699
5b-H
4.205
4.218
4.192
3a-H
3.543
5a-H
2.935
2.948
2.922
Ts-Me
2.551
2'-Me
2.444
0.94
2.01
2.00
0.94
1.06
2.06
1.90
1.96
1.86
2.98
2.97
Chemical Shift (ppm)

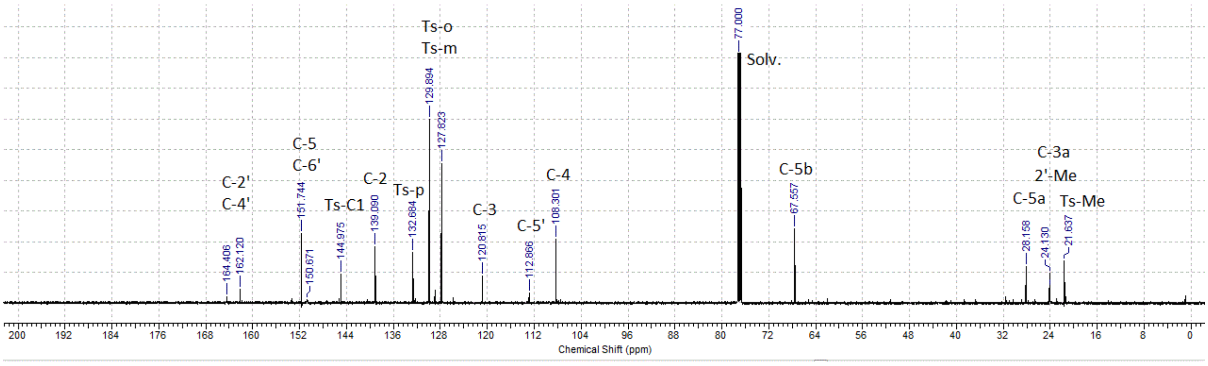
77.000
Solv.
Ts-o
Ts-m
129.894
127.823
C-5
C-6'
151.744
150.671
C-2'
C-4'
164.406
162.120
Ts-C1
144.975
C-2
139.090
Ts-p
132.684
C-3
120.815
C-5'
112.866
C-4
108.301
C-5b
67.557
C-3a
2'-Me
C-5a
Ts-Me
28.158
24.130
21.637
Chemical Shift (ppm)

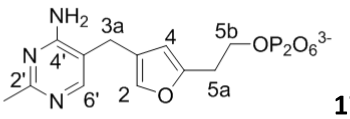

17 in D_2O

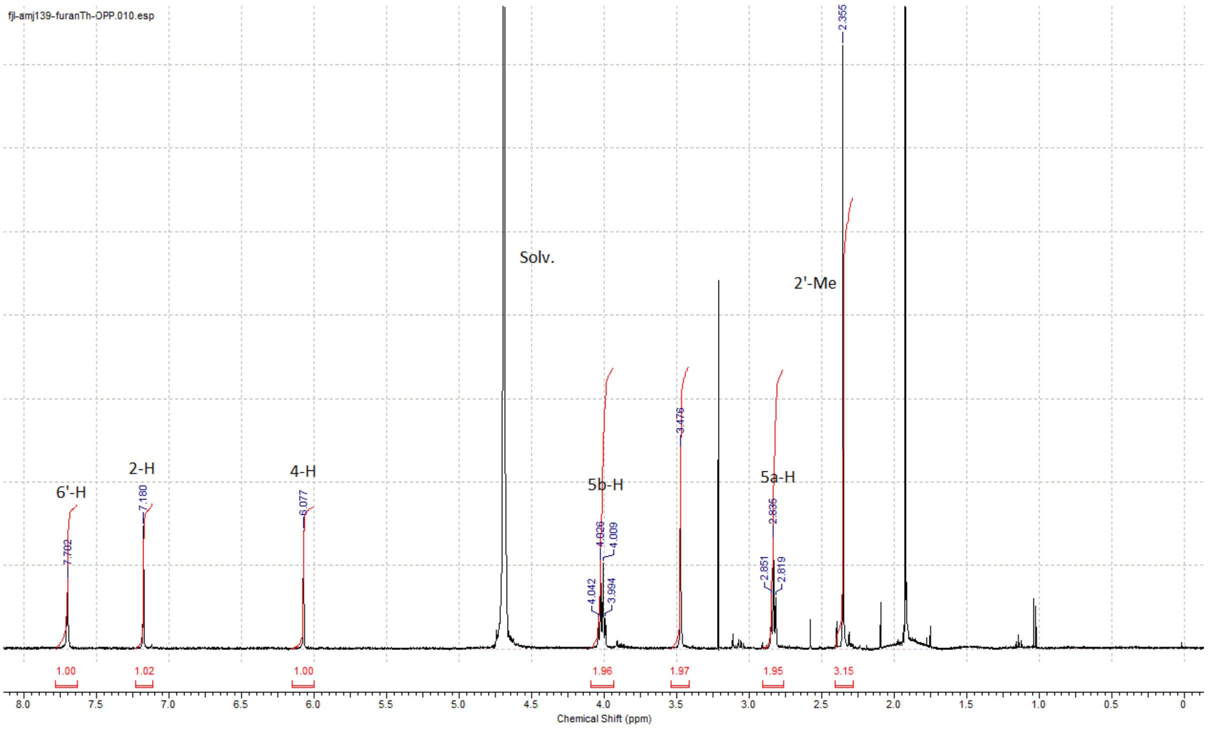

^{31}P NMR

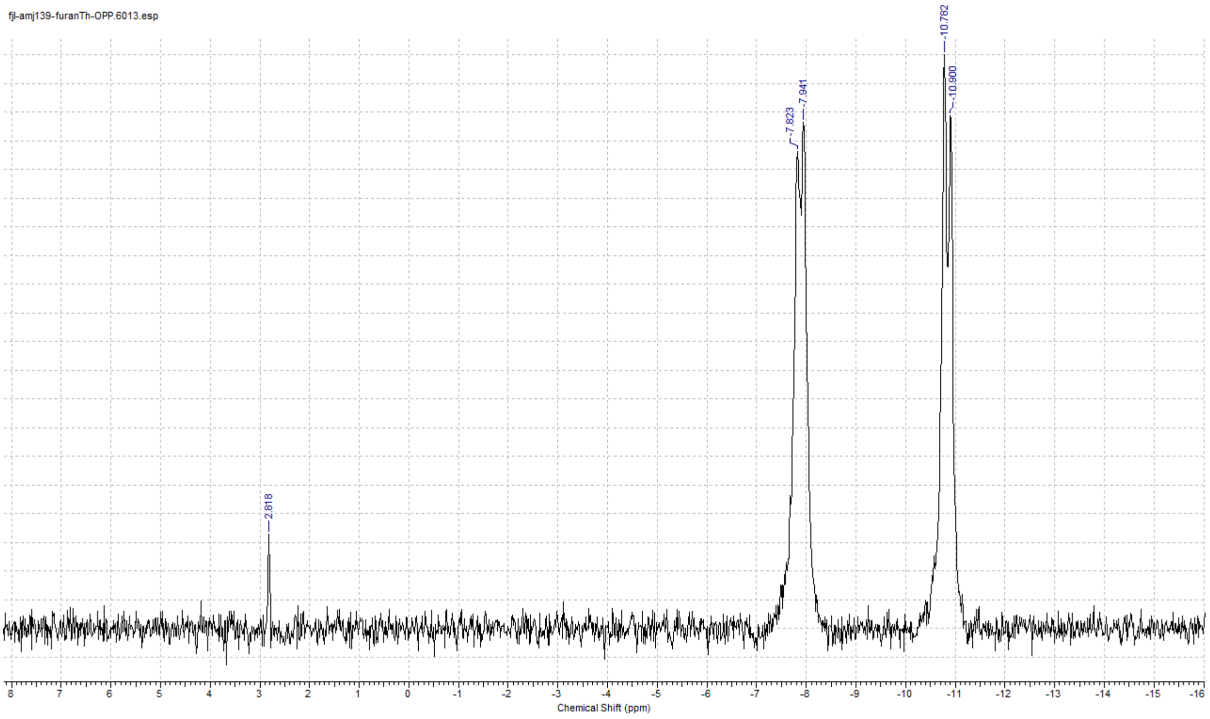

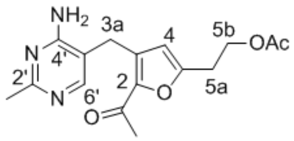

18 in CD_3OD

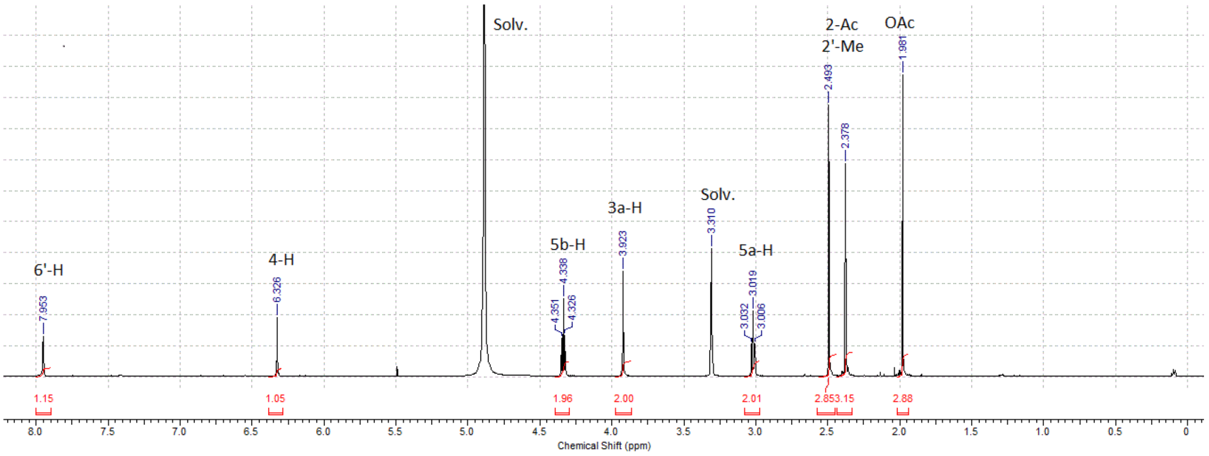

Solv.
2-Ac
2'-Me
OAc
Solv.
3a-H
5b-H
5a-H
4-H
6'-H
7.953
6.326
4.351
4.338
4.326
3.923
3.310
3.032
3.019
3.006
2.493
2.378
1.981
1.15
1.05
1.96
2.00
2.01
2.85
3.15
2.88
Chemical Shift (ppm)

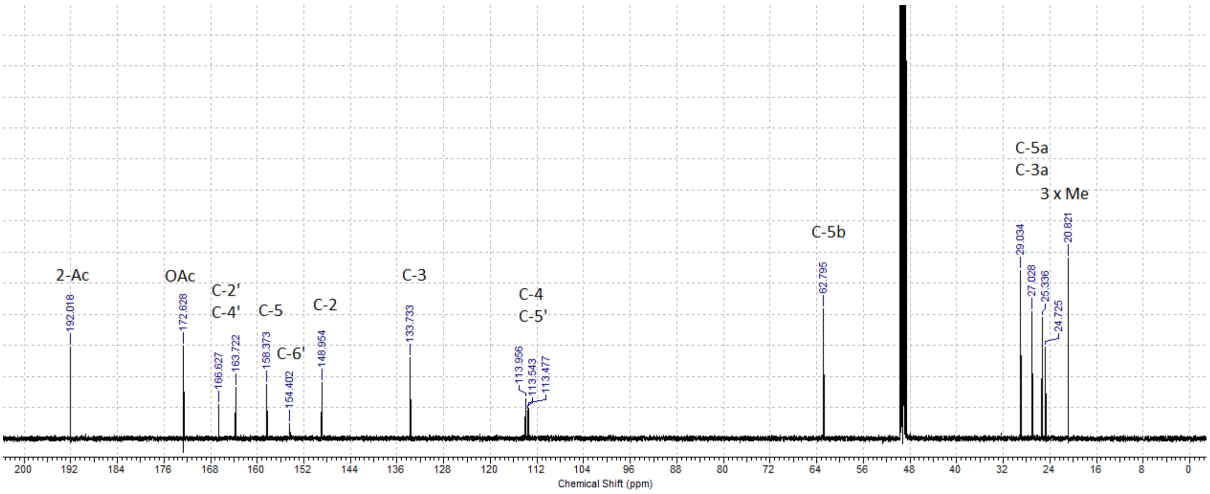

C-5a
C-3a
3 x Me
C-5b
2-Ac
OAc
C-2'
C-4'
C-5
C-6'
C-2
C-3
C-4
C-5'
192.018
172.628
166.627
163.722
158.373
154.402
148.954
133.733
113.956
113.543
113.477
62.795
29.034
27.028
25.336
24.725
20.821
Chemical Shift (ppm)

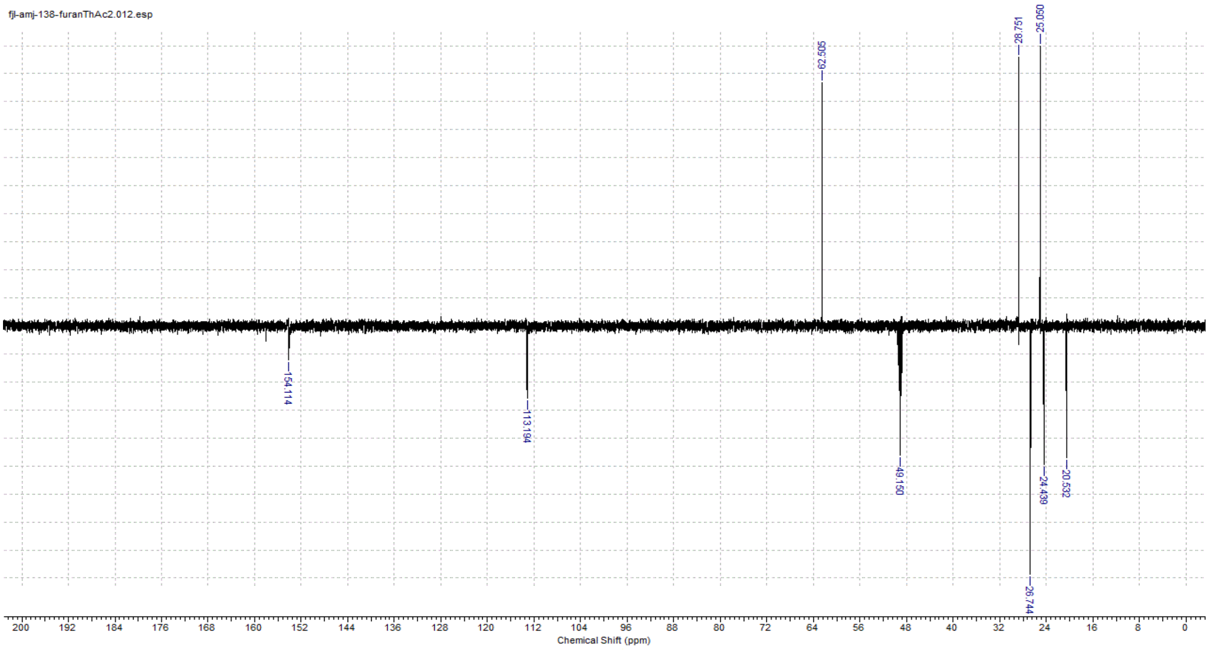
fjl-amj-138-furanThAc2.012.esp
62.505
28.751
25.050
154.114
113.194
49.150
24.439
20.532
26.744
Chemical Shift (ppm)

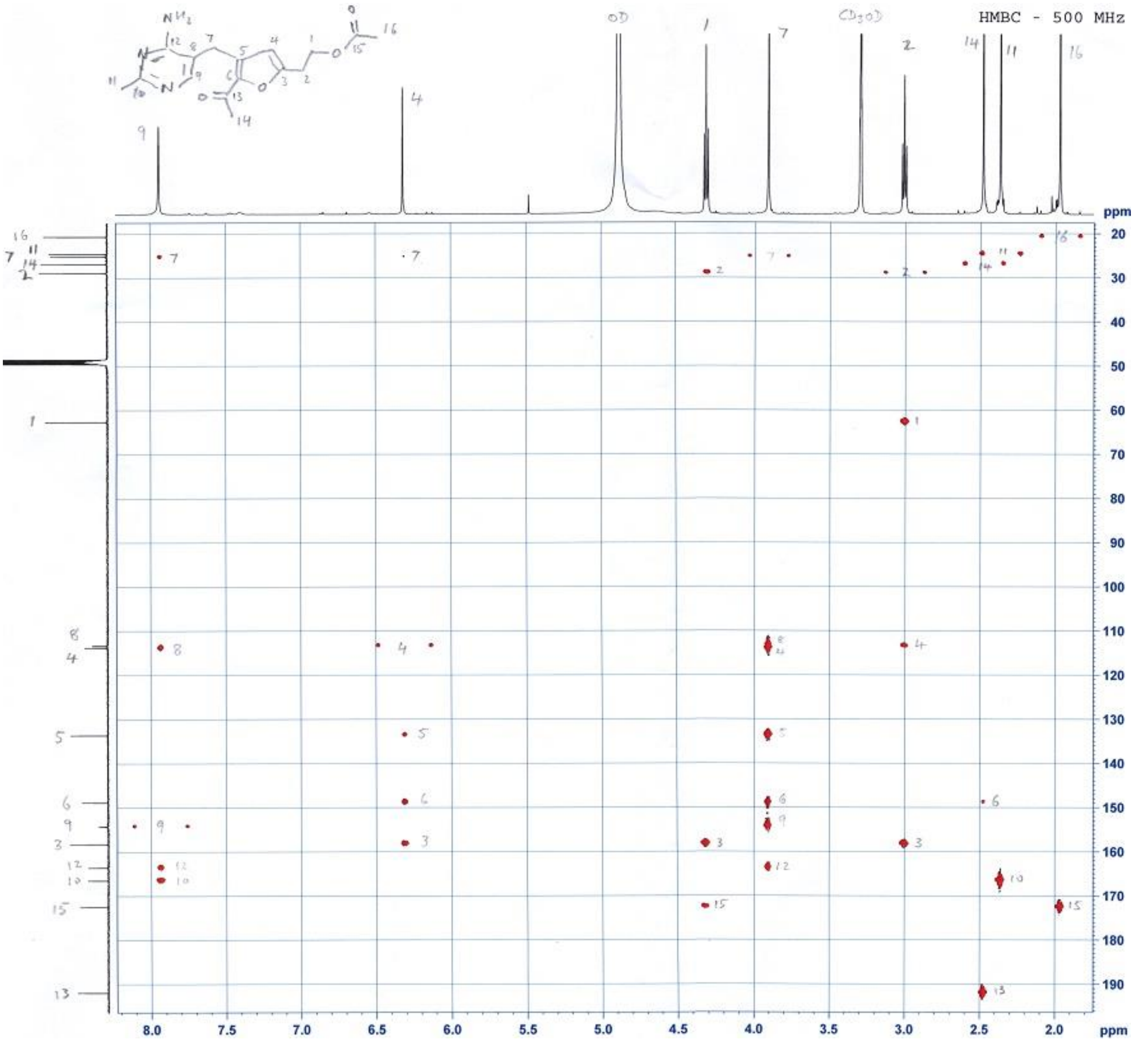
HMBC - 500 MHz
ppm

References

1. Ravindar, K.; Reddy, M. S.; Deslongchamps, P. *Org. Lett.* **2011**, *13*, 3178-3181.

2. Schenk, G.; Leeper, F. J.; England, R.; Nixon P. F.; Duggleby. R. G. *Eur. J. Biochem.,* **1997**, *248*, 63-71.

3. Gibson D. G.; Young L.; Chuang. R.-Y.; Venter. J. C.; Hutchison. C. A.; Smith. H. O. *Nature Methods* **2009**, *6*, 343-345.

4. Sambrook, J.; Fritsch, E. F.; Maniatis, T. *Molecular cloning: a laboratory manual*, 2nd ed. Cold Spring Harbor Laboratory Press, Cold Spring Harbor, New York, 1989.

5. Bradford, M. M. *Anal. Biochem.* **1976**, *72*, 248-254.

6. Mann, S.; Melero, C. P.; Hawksley, D.; Leeper, F. J. *Org. Biomol. Chem.* **2004**, *2*, 1732-1741.

7. Erixon, K. M.; Dabalos, C. L.; Leeper, F. J. *Org. Biomol. Chem.* **2008**, *6*, 3561-3572.
